# A long-acting formulation of rifabutin is effective for prevention and treatment of *Mycobacterium tuberculosis*

**DOI:** 10.1038/s41467-022-32043-3

**Published:** 2022-08-08

**Authors:** Manse Kim, Claire E. Johnson, Alan A. Schmalstig, Ayano Annis, Sarah E. Wessel, Brian Van Horn, Amanda Schauer, Agata A. Exner, Jason E. Stout, Angela Wahl, Miriam Braunstein, J. Victor Garcia, Martina Kovarova

**Affiliations:** 1 International Center for the Advancement of Translational Science, University of North Carolina at Chapel Hill, Chapel Hill, NC USA; 2 Division of Infectious Diseases, Department of Medicine, University of North Carolina at Chapel Hill, Chapel Hill, NC USA; 3 Center for AIDS Research, University of North Carolina at Chapel Hill, Chapel Hill, NC USA; 4 Department of Microbiology and Immunology, University of North Carolina at Chapel Hill, Chapel Hill, NC USA; 5 UNC Eshelman School of Pharmacy, University of North Carolina at Chapel Hill, Chapel Hill, NC USA; 6 Department of Radiology, Case Western Reserve University, Cleveland, OH USA; 7 Division of Infectious Diseases, Department of Medicine, Duke University, Durham, NC USA

**Keywords:** Drug delivery, Antibiotics, Tuberculosis

## Abstract

Tuberculosis (TB) is a communicable disease caused by *Mycobacterium tuberculosis* (*Mtb*) and is a major cause of morbidity and mortality. Successful treatment requires strict adherence to drug regimens for prolonged periods of time. Long-acting (LA) delivery systems have the potential to improve adherence. Here, we show the development of LA injectable drug formulations of the anti-TB drug rifabutin made of biodegradable polymers and biocompatible solvents that solidifies after subcutaneous injection. Addition of amphiphilic compounds increases drug solubility, allowing to significantly increase formulation drug load. Solidified implants have organized microstructures that change with formulation composition. Higher drug load results in smaller pore size that alters implant erosion and allows sustained drug release. The translational relevance of these observations in BALB/c mice is demonstrated by (1) delivering high plasma drug concentrations for 16 weeks, (2) preventing acquisition of Mtb infection, and (3) clearing acute Mtb infection from the lung and other tissues.

## Abstract (web summary)

Non-adherence to anti-tubercular therapy *Mycobacterium tuberculosis* infection can lead to treatment failure and even the development of drug resistance. In this work, the authors develop a long-acting delivery system of the anti-tuberculosis drug rifabutin and assess translational potential in vivo.

## Introduction

Tuberculosis (TB), which is caused by *Mycobacterium tuberculosis* (*Mtb)*^1^ is a world health concern with high morbidity and mortality^2^. An estimated 10 million people developed TB in 2020, resulting in 1.3 million deaths^2^. Moreover, approximately one-fourth of the world’s population has a latent TB infection (LTBI) with the potential for reactivation^2^. Of additional concern is the rise in drug-resistant *Mtb*^2^. Early diagnosis, treatment of all patients with TB, prevention of LTBI reactivation, and prevention of initial TB infection are all crucial to the WHO strategy in combating the TB epidemic^3^. TB preventative therapy with a single anti-TB drug, which acts to prevent initial infection or reactivation of LTBI, is highly effective and can reduce TB incidence when taken consistently^4^. For cases of active drug-susceptible TB disease, multidrug treatment regimens have an intensive phase of 2 months, followed by a continuation phase of at least 4 months^5^. Non-adherence to TB treatment can lead to treatment failure and the development of drug resistance^6^. Long-acting (LA) parenteral drug formulations that provide sustained drug release over weeks or months have the potential to reduce dosing frequency such that only one or two injections of the drug could be sufficient for TB treatment. This would dramatically change anti-TB treatment, as less frequent dosing could increase treatment compliance and consequently limit the occurrence of drug resistance^7–9^. LA bedaquiline for prevention of LTBI reactivation is currently being developed^10,11^. Affordable LA formulations with generic anti-TB drugs would allow the use of this approach in low-income communities.

LA biodegradable in situ forming implant (ISFI) formulations are attractive due to their unique properties which allow for subcutaneous administration of liquid formulations that solidify and form an implant at the site of injection. Injectable formulations are less invasive and less painful to administer than solid implants, and the biodegradable nature of the polymer matrix eliminates the need for surgical implant removal^12^. However, in the event of serious adverse reactions or at the completion of the treatment, the implant can be removed^13,14^. The removal of the implant stops drug delivery and prevents long-term exposure to subtherapeutic RFB concentrations^13,14^. To develop an ISFI LA formulation, the drug of interest and biodegradable polymer are solubilized in water miscible organic solvents^12^. Upon injection, phase transition occurs by solvent exchange, and polymer precipitation results in the formation of a solid implant consisting of biodegradable polymer and drug^12,14^. The drug release from the implant is controlled by implant structure^15,16^, polymer biodegradation^17^ and the composition of the liquid formulation^18^. Several ISFI formulations have been approved by the Food and Drug Administration (FDA) and include treatments for cancer, schizophrenia, and opioid dependency^19–24^. Eligard, Sublocade, and Perseris utilize biodegradable poly(lactic-co-glycolic) acid (PLGA) and N-methylpyrrolidone (NMP). Atridox contains biodegradable polylactic acid (PLA) and NMP. Onyx®Liquid Embolic system is a drug-free formulation based on ethylene vinyl alcohol co-polymer and dimethyl sulfoxide (DMSO) solvent. All formulations are injectable and form an implant after administration^20–24^.

Rifamycins, including rifampin (RIF), rifapentine (RFP), and rifabutin (RFB), are the cornerstones of TB therapy due to their potent bactericidal activity and their ability to inhibit DNA‐dependent RNA synthesis in prokaryotes^25^. RFB is a hydrophobic drug (LogP = 4.7) with reduced potential for drug-drug interactions, which makes it more suitable for treatment of *Mtb*/HIV coinfections compared to other rifamycins^26,27^. RFB also has higher tissue uptake due to its high lipophilicity, larger volume of distribution, longer terminal half-life, lower minimum inhibitory concentration (MIC) for *Mtb*, and higher tissue-to-plasma drug concentration ratio compared to RIF^26–29^. In humans, oral administration of 150 mg of RFB resulted in C_max_ 460 ng mL^−1^, C_min_ 50 ng mL^−1^, and tissue to plasma ratio of 5.6–6.8 in the lungs^30,31^. RFB is available as a low-cost generic medication and was selected as a model drug for development of a LA anti-TB drug formulation in this study. Multiple studies illustrate that RFB is safe and effective for treatment of active TB when patients have an adverse reaction to RIF, and RFB is particularly recommended instead of RIF for treatment of TB in patients with HIV utilizing certain antiretroviral therapies^32–34^.

**Fig. 1:**
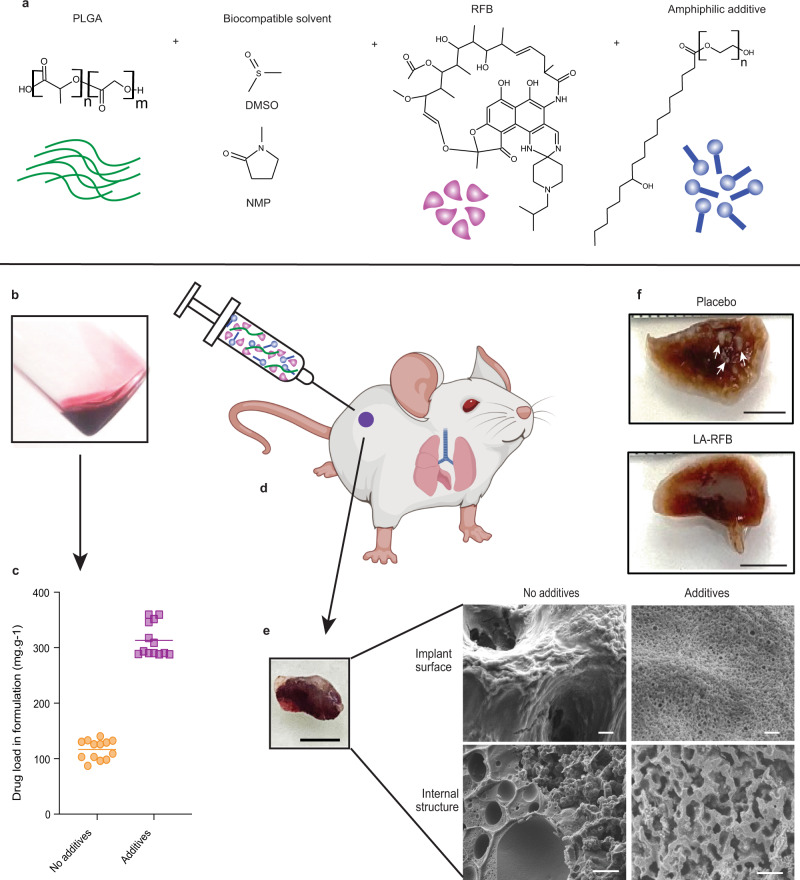
Critical changes in the material composition of in situ forming implant formulations result in structural changes, increased payload, reduced erosion, and long-term effective drug delivery. **a** Schematic showing LA-RFB composition consisting of PLGA as the biodegradable polymer, DMSO or NMP as a biocompatible solvent, RFB as an active pharmacological ingredient, and Kolliphor^®^HS 15 as an example of an additive. **b** The liquid injectable LA-RFB. **c** The drug load increases in LA-RFB formulations after addition of amphiphilic additives, *n* = 13 per group, mean value are shown, *P* = 0.0001, **d** The LA-RFB formulation can be administered subcutaneously and solidifies after injection. **e** A solidified implant of 50 μL LA-RFB, scale bar is 5 mm, and microphotographs of the implant surface and the internal structure without and without additives. Representative images are shown, three regions of each implant were scanned; three different implants were anlyzed for each formulation. Scale bar is 2 μm for the implant surface and 10 μm for the internal structure. **f** Formalin-fixed whole lung lobes of mice treated with placebo or LA-RFB prior to *Mtb* exposure. White lesions caused by *Mtb* are visible in lungs from mice which received placebo and are not present in mice which received LA-RFB (white arrows). Representative images of lung from six animals are shown. Parts of this figure were created using BioRender.com (2022). Source data for the panel **c** are provided as a Source data file.

ISFI LA formulations require a delicate composition balance to accommodate sufficient amounts of drug and polymer solubilized in biocompatible solvent. This severely limits the amount, number, and types of drugs that can be successfully formulated and represents a significant challenge when formulating RFB into an ISFI. Here, we show the development of LA-RFB injectable formulations that contain a biodegradable polymer (poly(lactic-co-glycolic-acid) (PLGA), a biocompatible water miscible solvent, RFB, and an amphiphilic additive (Fig. 1a, b) that dramatically increases drug load (Fig. 1c). After injection, these liquid formulations solidify in hydrophilic environments into an implant with porous microstructures, slow implant erosion and extended drug release (Fig. 1d, e). In vivo, a single subcutaneous injection of LA-RFB is able to deliver drug for 4 months, efficiently preventing *Mtb* infection after aerosol exposure, treating acute *Mtb* infection (Fig. 1f), and preventing *Mtb* dissemination to other organs.

## Results

### Amphiphilic additives enhance RFB solubility and improve LA-RFB release kinetics

LA-RFB formulations were prepared by dissolving RFB at the maximum solubility in DMSO or NMP (202 ± 22 and 148 ± 34 mg mL^−1^, respectively, mean ± SD). Biodegradable ester-capped PLGA polymer with different molecular weights (MW, 10.6 kDa, 22.9 kDa, or 36.8 kDa and an LA:GA ratio of 50:50) was added to the RFB solution at multiple solvent to polymer mass ratios (2:1, 4:1, and 5:1) (Table 1). The resulting formulations were evaluated for (1) their injectability, (2) implant formation after injection to aqueous release medium (PBS), (3) initial release burst, defined as a release of RFB (%) within the first 72 h of incubation in release medium, and (4) daily release rate (μg day^−1^) at 37 °C with 100 rpm shaking under sink conditions (concentration of RFB in release medium <42 μg mL^−1^) (Table 1).

**Table 1:** In vitro characteristics of LA-RFB formulations

Formulation	PLGA MW [kDa]	Solvent	Solvent: polymer ratio	Drug load [mg g^−1^]	Injectable^a^	Initial release burst [%]^b^	Release rate at 4 weeks [µg day^−1^]	Release rate at 8 weeks [µg day^−1^]	Release rate at 12 weeks [µg day^−1^]
RFB1	36.8	DMSO	4:1	128	Y	27.1 ± 6.3	2.4 ± 2.3	17.6 ± 4.4	16.1 ± 14.6
RFB2	36.8	DMSO	2:1	109	Y	11.8 ± 2.0	5.0 ± 1.1	17.6 ± 16.2	10.9 ± 12.6
RFB3	36.8	NMP	4:1	103	Y	32.2 ± 11.3	2.6 ± 3.0	23.7 ± 13.8	19.7 ± 13.3
RFB4	36.8	NMP	2:1	87	Y	16.0 ± 5.4	6.0 ± 2.3	9.5 ± 5.7	17.4 ± 12.6
RFB5	22.9	DMSO	4:1	128	Y	18.5 ± 2.5	16.3 ± 10.1	42.3 ± 25.9	3.9 ± 1.2
RFB6	22.9	DMSO	2:1	109	Y	12.0 ± 2.4	9.5 ± 8.4	19.7 ± 2.6	3.5 ± 0.6
RFB7	22.9	NMP	4:1	103	Y	24.2 ± 2.8	7.3 ± 5.0	42.9 ± 25.4	2.7 ± 4.7
RFB8	22.9	NMP	2:1	87	Y	11.5 ± 3.7	2.5 ± 0.3	36.2 ± 5.0	13.8 ± 6.2
RFB9	10.6	DMSO	4:1	127	Y	25.7 ± 5.5	69.2 ± 5.6	49.0 ± 25.9	5.9 ± 9.0
RFB10	10.6	DMSO	2:1	109	Y	13.0 ± 1.6	59.9 ± 11.8	31.8 ± 5.4	33.0 ± 7.0
RFB11^c^	10.6	NMP	4:1	103	Y	46.6 ± 11.0	22.3 ± 5.3	18.5 ± 21.5	N/A
RFB12	10.6	NMP	2:1	87	Y	19.5 ± 3.3	18.8 ± 4.5	14.2 ± 4.8	21.2 ± 7.7
RFB13^d^	10.6	DMSO	5:1	133	Y	22.7 ± 5.6	78.8 ± 18.9	18.2 ± 6.9	N/A

Data are expressed as mean ± SD, Source data are provided as a Source data file.

^a^Y = Injectable using a 1-mL syringe with a 19-gauge needle.

^b^Initial release burst is the cumulative percentage of RFB released into the release medium (PBS) 72 h after the injection.

^c^RFB11 implant was degraded after 9 weeks of incubation.

^d^RFB13 implant was degraded at 8 weeks incubation.

All 13 formulations were injectable, solidified after injection into PBS, and released RFB for over 8 weeks. Examples of release profiles and daily release rates are shown in Supplementary Fig. 1a, b. Release properties changed based on the composition of the individual formulation. Specifically, the initial release burst was higher in formulations with a 4:1 solvent to polymer ratio than in formulations with a 2:1 solvent to polymer ratio (Table 1). RFB daily release rates at 4 weeks of incubation were significantly higher in formulations with low MW PLGA compared to formulations with higher MW PLGA (Supplementary Fig. 1c). LA-RFB formulations with DMSO and a low MW PLGA (10.6 kDa) showed higher release rates at 4 weeks compared to formulations with NMP (Table 1, Supplementary Fig. 1d). These results demonstrate that by manipulating the composition of the ISFI formulation, release of drug from solidified implants can be modulated^14^.

**Fig. 2:**
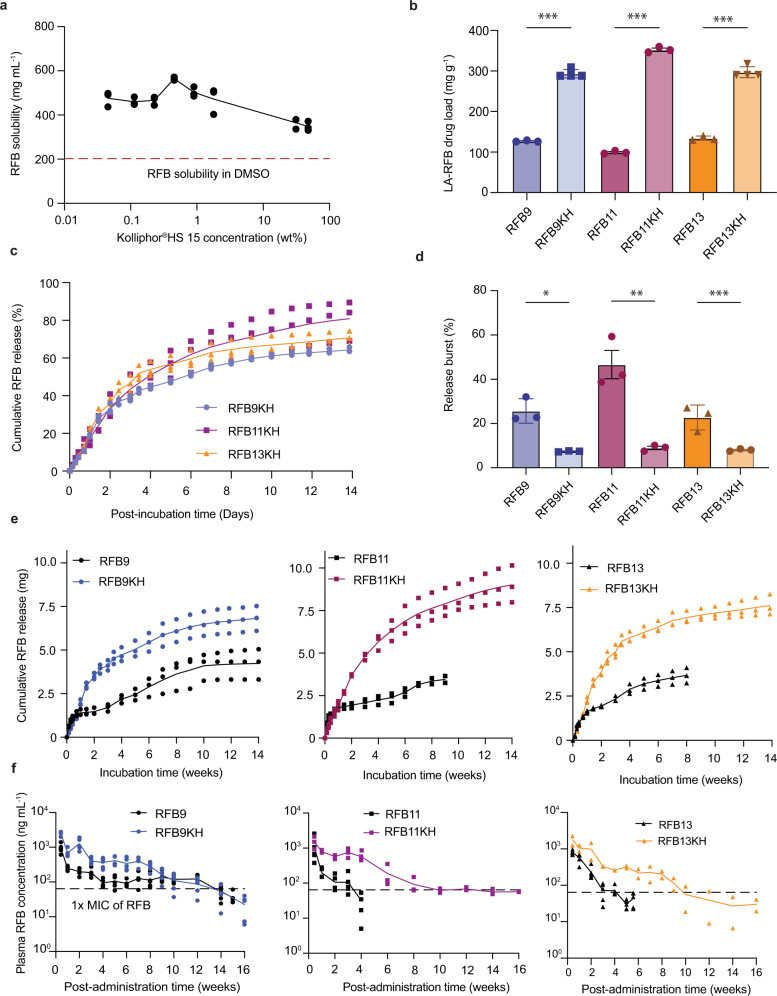
Amphiphilic additives increase drug load in LA-RFB ISFI formulations and extend the duration of drug release. **a** Saturated solubility of RFB in DMSO with different concentrations of Kolliphor^®^HS 15 (*n* = 3 per concentration indicated in the log x axis). Dashed line indicates RFB solubility in DMSO without the addition of Kolliphor^®^HS 15. **b** Comparison of drug loads in LA-RFB formulations with Kolliphor^®^HS 15 (RFB9KH, RFB11KH, RFB13KH) or without Kolliphor^®^HS 15 (RFB9, RFB11, RFB13); *n* = 3 per formulation. Mean ± SD is shown. ***P = 0.0001. **c** Cumulative in vitro RFB release (%) from ISFI formulations containing Kolliphor^®^HS 15; *n* = 3 per formulation; mean and individual values are shown. **d** Comparison of in vitro release bursts between LA-RFB formulations with and without Kolliphor^®^HS 15 (*n* = 3 per group, mean ± SD); *P = 0.0173, **P = 0.0001, ***P = 0.044. **e** In vitro cumulative RFB release (mg) from LA-RFB ISFI formulations with and without Kolliphor^®^HS 15, mean and individual values are shown, *n* = 3 per formulation. **f** RFB plasma concentration after a single subcutaneous injection (50 μL) into BALB/c mice of LA-RFB ISFI formulations with Kolliphor^®^HS 15 (RFB9, RFB11, RFB13) and without Kolliphor^®^HS 15 (RFB9KH, RFB11KH, RFB13KH). *n* = 5 (RFB9, RFB11, RFB13, RFB9KH), *n* = 3 (RFB11KH), and *n* = 2 (RFB13KH); mean and individual values are shown. Source data are provided as a Source data file.

Amphiphilic additives improve drug solubility in water by trapping molecules and forming nanoscale aggregates; micelles^35,36^. DMSO and NMP are polar solvents. The addition of Kolliphor^®^HS 15, an amphiphilic nonionic surfactant formerly known as Solutol HS 15, to DMSO dramatically increased RFB solubility (Fig. 2a). The highest RFB solubility, 564 ± 7 mg RFB per mL of DMSO (2.8 times higher than the RFB solubility in DMSO alone (202 ± 22 mg mL^−1^)) was achieved at 0.45% Kolliphor^®^HS 15. Similarly, the presence of Kolliphor^®^HS 15 in NMP also increased RFB solubility from 148 ± 34 mg mL^−1^ to 702 ± 10 mg mL^−1^ in NMP with 8.8% Kolliphor^®^HS 15 (Supplementary Fig. 2a). RFB solubility in DMSO was also substantially improved in the presence of low concentrations of other amphiphilic compounds, including D-α-Tocopherol polyethylene glycol 1000 succinate (TPGS, Supplementary Fig. 2b), Tween 20, Tween 80 (Supplementary Fig. 2c), and Pluronic F127 and F68 (Supplementary Fig. 2d).

To determine if the improved RFB solubility would result in increased RFB load in LA-RFB formulations, the composition of the formulations with the highest release rates at 4 weeks of incubation (RFB9, RFB11, and RFB13, Table 1) were modified to include 0.45% Kolliphor^®^HS 15 in the DMSO-based formulations (RFB9 and RFB13) and 8.8% Kolliphor^®^HS 15 in the NMP-based formulation (RFB11). To indicate the presence of Kolliphor^®^HS 15 and a high drug load, new formulations were denoted RFB9KH, RFB11KH, and RFB13KH (Supplementary Table 1). The RFB load in formulation RFB9KH increased from 126.9 ± 1.8 mg g^−1^ (RFB9) to 293.4 ± 10.2 mg g^−1^, the RFB load in formulation RFB11KH increased from 99.1 ± 3.6 mg g^−1^ (RFB11) to 352.4 ± 6.8 mg g^−1^, and the RFB load in formulation RFB13KH increased from 134.3 ± 5.3 mg g^−1^ (RFB13) to 297.1 ± 13.5 mg g^−1^ (mean ± SD, Fig. 2b). Each of the new formulations (RFB9KH, RFB11KH, and RFB13KH) released RFB for at least 14 weeks in vitro (Fig. 2c). Notably, these formulations had a reduced initial release burst at 72 h (Fig. 2d). Specifically, RFB9KH had an initial burst 3.5 times lower than RFB9 (*P* = 0.0173), RFB11KH had an initial release burst 5.2 times lower than RFB11 (*P* = 0.0001), and RFB13KH had a release burst 2.8 times lower than RFB13 (*P* = 0.044, Fig. 2d). Consistent with their increased drug load, formulations containing Kolliphor^®^HS 15 also resulted in an increased amount of RFB released (Fig. 2e). Cumulative release of RFB in vitro after 8 weeks was 6104 ± 652 μg RFB from RFB9KH compared to 3630 ± 848 µg from RFB9 (*P* = 0.0160), 7639 ± 835 μg from RFB11KH compared to 3352 ± 247 μg from RFB11 (*P* = 0.0014), and 6930 ± 356 μg RFB from RFB13KH compared to 3667 ± 430 µg RFB from RFB13 (*P* = 0.0005).

BALB/c mice were administered a single subcutaneous injection (50 μL) of the indicated LA-RFB formulations and drug levels were monitored in plasma over time. Plasma RFB concentrations were above the RFB MIC (64 ng mL^−1^)^37^ for longer periods of times in all the mice treated with the formulations containing Kolliphor^®^HS 15. The most remarkable differences were noted with formulations RFB11KH and RFB13KH that released drug for 6 and 9 weeks longer than the corresponding formulations without Kolliphor^®^HS 15 (Fig. 2f). However, even though the duration of drug delivery from formulation RFB9KH was only extended by an additional 2 weeks, this formulation maintained RFB plasma concentrations above the MIC for the longest time and was therefore used for further optimization.

### Uncapped acid-ending PLGA increases RFB release rates at later stages of RFB delivery

**Fig. 3:**
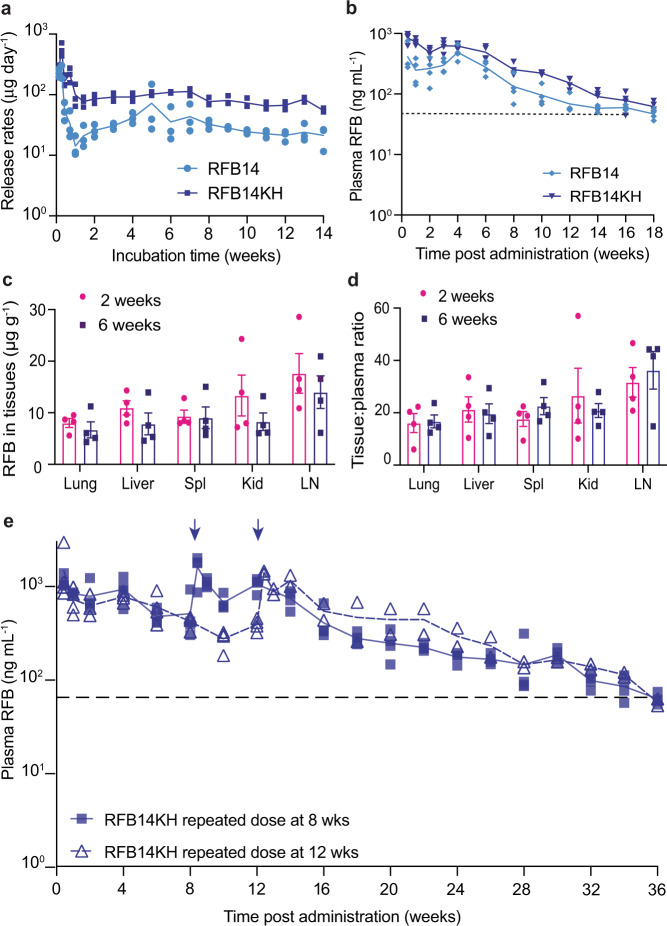
Improved RFB release by formulations containing uncapped acid-ending PLGA. **a** Daily in vitro RFB release from LA-RFB formulations containing acid-ending PLGA (LA:GA 50:50, MW 13.5 kDa) without Kolliphor^®^HS 15 (RFB14) or with Kolliphor^®^HS 15 (RFB14KH), *n* = 3 per formulation. **b** In vivo RFB plasma concentrations in BALB/c after a single subcutaneous injection (50 μL) of RFB14 or RFB14KH (*n* = 4). The dotted line indicates RFB MIC. **c** RFB tissue concentrations at 2 and 6 weeks post administration of RFB14KH (50 μL) in BALB/c mice; *n* = 4 per time point, mean ± SEM. **d** Tissue to plasma ratio of RFB concentrations at 2 and 6 weeks post administration of RFB14KH (*n* = 4 per time point, mean ± SEM). Spl: spleen, Kid: kidney, LN: lymph nodes. **e** Pharmacokinetics of RFB after a single subcutaneous administration of LA-RFB (50 μL) in BALB/c mice followed by a second injection of RFB14KH (50 μL) at 8 weeks (*n* = 4) or 12 weeks (*n* = 2) after the first dose. Mean and individual datapoints are shown; arrows indicate the time point of the booster injections, and the dotted line indicates RFB MIC. Source data are provided as a Source data file.

Terminal stages of drug release from PLGA implants are primarily controlled by polymer biodegradation^38^. A delay in degradation time has been found for an ester end-capped PLGA in comparison with a more hydrophilic PLGA without ester capping (acid-ending) of a similar molecular weight and co-polymer composition^39^. We therefore hypothesized that the use of uncapped acid-ending PLGA instead of ester end-capped PLGA would lead to increased release rates in vitro and higher drug concentrations at later time points. A new formulation, RFB14, was prepared with the same composition as RFB9, but with acid-ending PLGA (Supplementary Table 1). The RFB14 formulation demonstrated increased in vitro release rates at later time points compared to RFB9. For example, at 12 weeks RFB14 release rates were 24.5 ± 5.2 μg per day, compared to RFB9 release rates of 5.9 ± 9.0 μg per day (mean ± SD, Supplementary Table 1). Similarly, RFB14KH (a formulation with the same composition as RFB9KH but with an acid-ending polymer) also had a higher in vitro release rate at 12 weeks compared to RFB9KH (RFB14KH: 67.2 ± 5.7 μg per day, RFB9KH: 10 ± 2.6 μg per day, mean ± SD). RFB14KH had an increased release rate at all time points measured compared to RFB14 (Fig. 3a). Based on these encouraging results, we performed a pharmacokinetic analysis of these formulations in vivo using BALB/c mice. A single subcutaneous injection of 50 μL of RFB14 or RFB14KH showed that the formulations based on an acid-ending PLGA delivered higher RFB plasma concentrations compared to RFB9 and RFB9KH (Fig. 3b, Fig. 2f). At 16 weeks post administration, plasma RFB concentrations were three times higher in mice that received RFB14KH (78.1 ± 13.6 ng mL^−1^, mean ± S.E.M.) compared to mice administered RFB9KH (22.7 ± 6.8 ng mL^−1^, mean ± S.E.M.). Importantly, no visual changes in skin caused by adverse response to implant were noted throughout the experiment. During removal of residual implants in the end of experiment, no overt inflammatory reactions such as angiogenesis, strong fibrosis, or abscesses in the skin or other tissues in close proximity of placebo or drug-containing implants were noted.

The efficiency of RFB penetration into tissues was assessed two and six weeks after a single 50 μL subcutaneous injection of RFB14KH. RFB concentrations in organs susceptible to *Mtb* were analyzed by liquid chromatography followed by mass spectrometry (LC/MS/MS) (Fig. 3c). Consistent with sustained release of drug from the formulation, no differences were observed in RFB tissue concentrations analyzed at 2 and 6 weeks after RFB14KH administration (2 weeks: lung 8.0 ± 0.8 μg g^−1^, liver 11.0 ± 1.4 μg g^−1^, spleen 9.3 ± 1.2 μg g^−1^, kidney 13.3 ± 4.0 μg g^−1^, lymph nodes 17.6 ± 3.8 μg g^−1^; 6 weeks: lung 6.7 ± 1.6 μg g^−1^, liver 7.8 ± 2.1 μg g^−1^, spleen 9.0 ± 2.1 μg g^−1^, kidney 8.3 ± 1.7 μg g^−1^, lymph nodes 14.0 ± 3.2 μg g^−1^; data are expressed as mean ± S.E.M., lung *P* = 0.4951, liver *P* = 0.2000, spleen *P* = 0.8857, kidney *P* = 0.2000, lymph nodes *P* = 0.8857, Mann–Whitney U test). The mean tissue to plasma ratio at 2 weeks post administration was 16.4 in the lung, 20.5 in the liver, 20.1 in the spleen, 23.8 in the kidney, and 34.0 in the lymph nodes and was similar to the tissue to plasma ratio at 6 weeks post administration in tested organs (lung *P* = 0.9999, liver *P* = 0.8857, spleen *P* = 0.4857, kidney *P* = 0.9999, lymph nodes *P* = 0.8857, Mann–Whitney U test), suggesting high RFB penetration to tissues most affected by *Mtb* infection (Fig. 3d). In conclusion, acid-ending PLGA formulations had superior in vitro and in vivo properties compared to formulations with ester-capped PLGA, resulting in long-term drug release with substantial tissue penetration.

### A second injection of RFB14KH administered 8 or 12 weeks later provides drug delivery for up to 36 weeks

To extend RFB delivery beyond 4 months, in a separate experiment, a second dose of the same volume and composition of RFB14KH was administered 8 or 12 weeks after the first injection, and plasma levels of RFB were monitored longitudinally (Fig. 3e). All mice showed similar plasma RFB concentrations after the first dose of RFB14KH (compare panels b and e in Fig. 3). The booster injection rapidly increased plasma RFB concentrations back to the initial levels observed with the first dose, regardless of the timing of the booster. While mice that received one dose of RFB14KH showed plasma RFB concentrations that gradually decreased over time to the RFB MIC by 18 weeks post administration (Fig. 3b), mice that received a booster maintained RFB plasma concentrations above the MIC for 36 weeks (Fig. 3e).

### Long-term RFB stability in LA-RFB

The stability of the RFB14KH formulation was evaluated during storage at room temperature (25 °C) in the dark for changes in physical appearance, residual RFB concentration, and chemical integrity at 4, 6, 9, 12, 15, and 18 months. The residual RFB compared to the initial RFB concentration in RFB14KH was 100.7 ± 7.4% at 4 months, 99.0 ± 0.4% at 6 months, 100.4 ± 0.3% at 9 months, 96.5 ± 1.0% at 12 months, 96 ± 0.5% at 15 months, and 98.2 ± 4.5% at 18 months (mean ± SD) (Supplementary Fig. 3a). HPLC histograms of the residual amount of RFB in RFB14KH showed a single peak with similar retention times (5.4–5.7 min) for 18 months of storage (Supplementary Fig. 3b). No change in physical appearance or consistency was observed. Interestingly, a solution of RFB in DMSO without polymer stored under the same conditions as RFB14KH had 67% residual RFB after 3 months, 53% after 4 months, and 25% after 6 months of storage, suggesting faster degradation of RFB in DMSO compared to RFB14KH (Supplementary Fig. 3c). These results indicate that the RFB14KH formulation is stable at room temperature for at least 18 months.

### Increased drug load improves RFB release kinetics from LA-RFB formulations

**Fig. 4:**
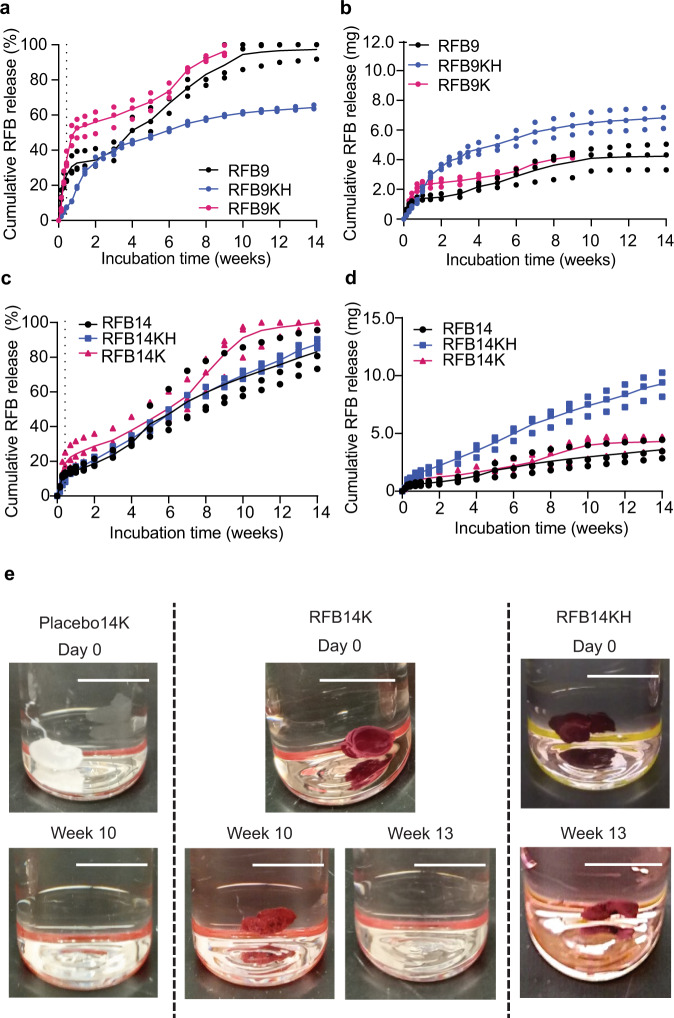
Impact of Kolliphor^®^HS 15 and increased drug load on RFB release kinetics from ISFI formulations. **a**–**d** In vitro evaluation of formulations RFB9 (127 mg g^−1^ RFB) and RFB14 (130 mg g^−1^ RFB) with formulations with high drug load and 0.45 wt% Kolliphor^®^HS 15 (RFB9KH: 293 mg g^−1^ RFB, RFB14KH: 294 mg g^−1^ RFB), and formulations containing Kolliphor^®^HS 15 and the same drug load as RFB9 and RFB14 (RFB9K: 128 mg g^−1^ RFB, RFB14K: 132 mg g^−1^ RFB) (*n* = 3 per formulation). Cumulative RFB release in % over time of RFB9, RFB9KH, RFB9K (**a**) and RFB14, RFB14KH, RFB14K (**c**). Cumulative RFB release (μg) over time of RFB9, RFB9KH, RFB9K (**b**) and RFB14, RFB14KH, RFB14K (**d**). **e** Representative images of a placebo implant with 0.45 wt% Kolliphor^®^HS 15 but without RFB (placebo14K), an implant with 132 mg g^−1^ RFB load and Kolliphor^®^HS 15 (RFB14K), and an implant with 294 mg g^−1^ RFB load and Kolliphor^®^HS 15 (RFB14KH) in PBS (pH 7.4) over time. Scale bar = 1 cm. *n* = 3 implants per formulation. Source data for panels **a**–**d** are provided as a Source data file.

To distinguish whether Kolliphor^®^HS 15 or the increased drug load contributed to the improved RFB release kinetics, new LA-RFB formulations were prepared. Formulations RFB9K and RFB14K had the same compositions and drug loads as formulations RFB9 and RFB14, respectively, but contain Kolliphor^®^HS 15. Therefore, any differences observed between formulations RFB9K and RFB9 (128 mg g^−1^ and 127 mg g^−1^ RFB, respectively) or RFB14K and RFB14 (132 mg g^−1^ and 130 mg g^−1^, respectively) that have the same drug load can be attributed to the presence of Kolliphor^®^HS 15. In addition, any differences observed between formulations RFB9K and RFB9KH (128 mg g^−1^ and 293 mg g^−1^, respectively) or RFB14K and RFB14KH (132 mg g^−1^ and 294 mg g^−1^, respectively) can be attributed to differences in RFB load. RFB9K demonstrated a high initial release burst (34.4 ± 4.5%, mean ± SD) compared to RFB9 (25.7 ± 5.5%, *P* = 0.0043) and RFB9KH (7.4 ± 0.2%, *P* = 0.0002, one-way ANOVA) (Fig. 4a). RFB9K released a similar amount of RFB to RFB9 over time but less than RFB9KH (Fig. 4b). Specifically, at 8 weeks of in vitro incubation, cumulative release of RFB from RFB9K was 4218 ± 135 μg, RFB9 3860 ± 933 μg (*P* = 0.7242), and RFB9KH 6328 ± 681 μg of RFB (mean ± SD, *P* = 0.0148, RFB9KH compared to RFB9K; *P* = 0.0065, RFB9KH compared to RFB9, one-way ANOVA). RFB14K showed a similar initial release burst of RFB compared to RFB14 that was higher when compared to the release rate of RFB14KH (18.8 ± 5.7%, 9.6 ± 1.6%, and 12.6 ± 0.9%, respectively; Fig. 4c and Supplementary Table 1, *P* = 0.0406, RFB14K compared to RFB14KH, one-way ANOVA). After 12 weeks of incubation in PBS, cumulative release of RFB from RFB14KH was higher compared to RFB14K (*P* = 0.002) and RFB14 (*P* = 0.003) (RFB14: 3753 ± 924 μg, RFB14K: 3274 ± 50 μg, RFB14KH: 8371 ± 616 μg, one-way ANOVA) (Fig. 4d). These results indicate that the addition of Kolliphor^®^HS 15 to the formulation increased the initial release burst, while the high drug load in the formulation resulted in sustained and increased RFB release over a long period of time.

### Increased drug load decreases implant erosion

To further investigate the basis for the extended release from formulation RFB14KH, we evaluated implant erosion in vitro. Interestingly, we noted that implant erosion in vitro decreased inversely to the drug load present in the implant. Placebo14K with acid-ending PLGA and Kolliphor^®^HS 15 had the same composition as RFB14KH but did not contain RFB (placebo14K, 13.5 kDa acid-ending PLGA, 0.45 wt% Kolliphor^®^HS 15 in DMSO, 4:1 DMSO:PLGA ratio). Placebo14K formed implants that took 10 weeks to fully dissipate in vitro (Fig. 4e). Implants formed from RFB14K that had the same composition as placebo14K and contained 132 ± 6 mg g^−1^ RFB completely dissipated by 13 weeks of incubation in PBS (Fig. 4e). Notably, implants formed from RFB14KH with 294 mg g^−1^ RFB were not eroded by 13 weeks (last time evaluated). Dissipation results suggest that higher drug load in the implant result in slower polymer erosion allowing for prolonged release of RFB from the implant.

### Effect of Kolliphor®HS 15 and drug load on LA-RFB implant structure

**Fig. 5:**
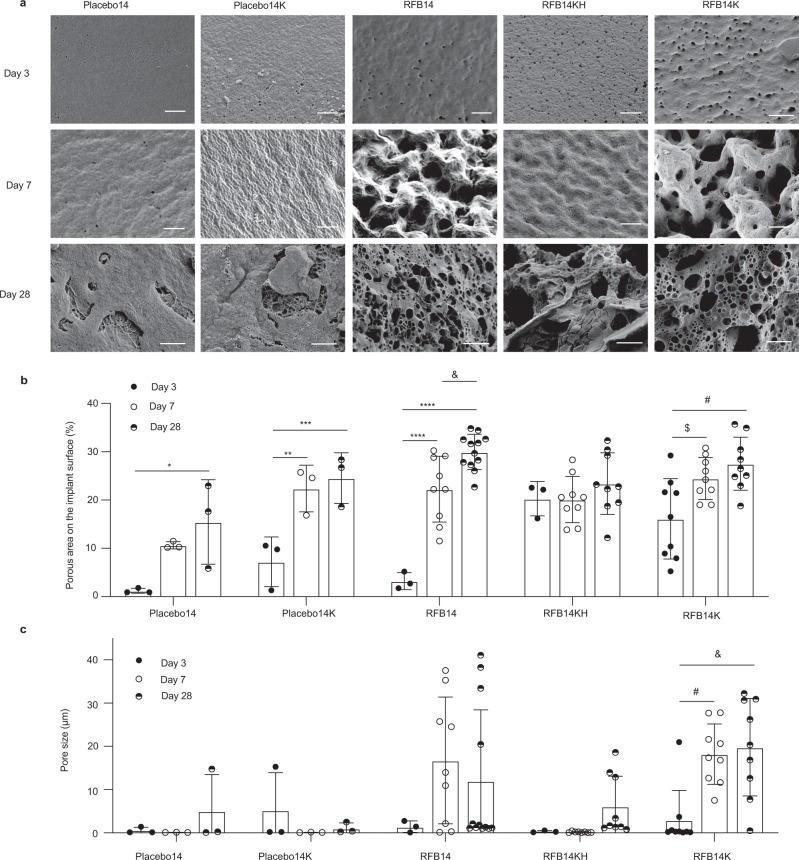
Surface morphology of LA-RFB implants depends on implant composition. **a** Representative SEM images of the surface of implants formed by RFB14, RFB14KH, and RFB14K and their corresponding placebos (placebo14 and placebo14K) in PBS after 3, 7, and 28 days of incubation. Scale bar = 10 μm. Representative images are shown, three regions of each implant was scanned, three different implants were analyzed for each time point and formulation. **b**, **c** Quantitative SEM analysis of implant surface images for changes in **b** porous area (*P = 0.032, **P = 0.024, ***P = 0.0136, ****P = 0.0001, ^&^*P* = 0.056, ^$^*P* = 0.0242, ^#^*P* = 0.0021) and **c** pore size (^#^*P* = 0.0027, ^&^*P* = 0.001) over time. Data are presented as mean values +/−SD. Source data for panels **b** and **c** are provided as a Source data file.

The kinetics of drug release from ISFI formulations are influenced by implant structure^16,40^ which is determined by multiple factors, including the composition of the formulation (polymer type, solvent, additives, and drug properties), the rate of phase inversion, the injection site, and polymer degradation^17,18,41,42^. Biodegradable ISFI polymer implants have a porous microstructure with an interconnected network of pores that allows diffusion of water into the implant and its bulk erosion. Pore size and the porous area on the surface of the implant determine water access and its uptake facilitating polymer degradation and implant erosion^43–45^. To further understand the basis for the improved release kinetics of RFB14KH, both surface and freeze fractured cross-sections of implants with different RFB load and composition were analyzed using scanning electron microscopy (SEM)^16^. Solidified implants of RFB14 (130 mg g^−1^ RFB), RFB14K (132 mg g^−1^ RFB), RFB14KH (294 mg g^−1^ RFB), placebo14 with the same composition as RFB14 but without drug (13.5 kDa acid-ending PLGA, 4:1 DMSO:PLGA ratio), and placebo14K with the same composition as RFB14KH and RFB14K but without RFB (Supplementary Table 1) were collected after 3 days of incubation in PBS (i.e., the end of the solidification process^14^) and after 7 and 28 days of incubation in PBS. SEM images of the implant surface (Fig. 5a, Supplementary Fig. 4a for higher magnification) were evaluated for porous area (defined as % of surface area filled with pores) (Fig. 5b) and pore size (μm) (Fig. 5c). Both placebo14 and placebo14K had low porous area (1.2 ± 0.5% and 7.2 ± 5.1%, respectively) that increased significantly over time (day 7: 10.6 ± 0.7% and 22.4 ± 4.8%, day 28: 15.5 ± 8.8% and 24.5 ± 5.2%, respectively) (Fig. 5b). Placebo implants had small pores (0.6 ± 0.7 μm and 5.2 ± 8.7 μm, respectively) which did not change during incubation (Fig. 5c). Pore sizes were similar between the placebo implants with and without Kolliphor^®^HS 15.

RFB14 implants with 130 mg g^−1^ RFB load and no Kolliphor^®^HS 15 had a low porous area (3.2 ± 1.8%) that significantly increased over time (22.2 ± 6.8% at 7 days and 30.0 ± 3.6% at 28 days, Fig. 5b). The presence of Kolliphor^®^HS 15 in LA-RFB formulations increased the initial porous area (RFB14KH: 20.3 ± 3.6% and RFB14K: 16.1 ± 9.7% vs. RFB14: 3.2 ± 1.8%). Implants with high RFB load (RFB14KH, 294 mg g^−1^ RFB) had no significant increase in porous area during the incubation period (RFB14KH: 20.3 ± 3.6% at 3 days, 20.1 ± 4.8% at 7 days, and 23.4 ± 6.4% at 28 days). These data indicate that the presence of Kolliphor^®^HS 15 in the formulation results in increased porous area on the implant surface after solidification (indicated as day 3 in panel b, Fig. 5). In addition, these data also indicate that increased drug load prevents further increases in porous area during incubation (indicated as days 7 and 28 in panel b, Fig. 5). Pore size on the surface of RFB14 and RFB14K implants increased during implant erosion (from 1.4 ± 1.3 μm and 3.0 ± 6.8 μm at 3 days to 16.7 ± 14.7 μm and 18.2 ± 7.0 μm at 7 days, and 29.6 ± 4 μm and 27.5 ± 5.5 μm at 28 days, mean ± SD). These increases in pore size did not occur during the first 7 days for the implants with high drug load and were significantly less after 28 days of incubation (RFB14KH: pore size 0.3 ± 0.2 μm at 3 days, 0.2 ± 0.1 μm at 7 days, and 6.1 ± 7.0 μm at 28 days of incubation, Fig. 5c). These results suggests that a high drug load slows implant erosion and stabilizes the implant, contributing to extended drug release.

The internal structure of LA-RFB implants had a typical honeycomb-like structure with uniform macro-voids and interconnected pores after 7 days of incubation (Supplementary Fig. 4b)^18^. Addition of Kolliphor^®^HS 15 resulted in increased asymmetry and disorganization of the internal implant structure (RFB14 vs. RFB14K). High drug load in the implant resulted in a more organized internal structure, with fewer macro-voids and interconnected pores (RFB14K vs. RFB14KH at 7 days of incubation). The internal structure of the implants for all LA-RFB formulations (RFB14, RFB14KH, and RFB14K) became less organized over time (Supplementary Fig. 4b). Cross-sections of the LA-RFB implants showed a defined shell at the implant surface with a more dense and organized structure than the internal structures of the implant. The shell was most distinct in implants with high drug load (RFB14KH), where it had highly organized fingerlike pores directly connecting the surface and internal parts of the implant (Supplementary Fig. 4b)^46^. Shells of implants with the same composition but with lower drug load were less organized than in implants with higher drug load, regardless of the presence of Kolliphor^®^HS 15 (RFB14 and RFB14K). By 28 days of incubation, the shells eroded and were no longer visible for all LA-RFB implants. Together, these results show that the presence of Kolliphor^®^HS 15 in LA-RFB formulations resulted in a large porous surface area after implant formation that facilitates water diffusion into the implant and faster polymer degradation and erosion of the implant^43^. High drug load slowed implant erosion despite the presence of Kolliphor^®^HS 15. Therefore, drug load in formulations containing Kolliphor^®^HS 15 has a large impact on modulating RFB release kinetics.

### In vivo efficacy of LA-RFB against *Mycobacterium tuberculosis*

**Fig. 6:**
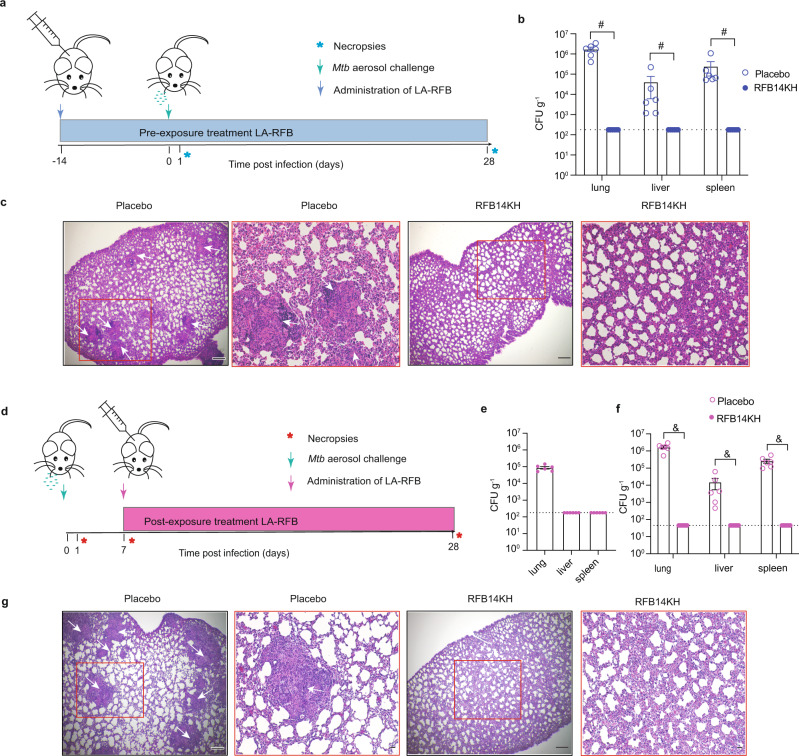
In vivo analysis of the efficacy of RFB14KH against *Mycobacterium tuberculosis* infection. **a** Experimental schema for pre-exposure treatment efficacy testing of LA-RFB. BALB/c mice were untreated (*n* = 4) or treated with a single subcutaneous injection of placebo (*n* = 6) or RFB14KH (*n* = 6) formulations. Two weeks after treatment, mice were exposed to *Mtb*. Control untreated mice were used to determine the dose of exposure 24 h after infection (*n* = 4). ISFI treated mice were analyzed for *Mtb* infection 4 weeks post-exposure. **b** Bacterial burden in lung, liver, and spleen of placebo (*n* = 6) and RFB14KH (*n* = 6) treated mice 4 weeks after *Mtb* exposure, mean +/−SEM are shown (^#^*P* = 0.0022 Mann–Whitney U test). **c** H&E staining of lung sections shows pathological changes in mice treated with placebo but not in mice treated with RFB14KH. Representative sections from 6 placebo and 6 RFB14KH treated mice are shown **d** Experimental schema for post-exposure LA-RFB treatment. BALB/c mice (*n* = 22) were exposed to *Mtb*; 24 h later, a group of 4 was analyzed to determine the exposure dose. *Mtb* infection was assessed again 1-week post exposure in a group of 6 mice to determine the levels of infection. At this time the remaining infected mice were treated with placebo (*n* = 6) or RFB14KH (*n* = 6). Animals were harvested and analyzed for *Mtb* infection 3 weeks later (4 weeks post exposure to *Mtb*). **e** Bacterial burden in mice 7 days post *Mtb* exposure (*n* = 6). **f** Bacterial burden 4 weeks post *Mtb* exposure, mean +/− SEM are shown (*n* = 6 per group, ^&^*P* = 0.0022, Mann–Whitney U test). **g** H&E staining of lung sections show pathological changes in mice treated with placebo but not in mice treated with RFB14KH. Representative sections from 6 placebo and 6 RFB14KH treated mice are shown. Scale bars in H&E images in **c** and **g** are 300 μm in low magnification images and 50 μm in higher magnification images. Dotted lines in panels **b**, **e**, and **f** indicate the limit of detection. Arrows in panels **c** and **g** indicate lesions caused by *Mtb*. Source data for panels **b**, **e**, and **f** are provided as a Source data file and in Supplementary Tables 2, 3, and 4.

The optimized LA-RFB formulation (RFB14KH) was tested in two in vivo experiments to determine its efficacy against *Mtb* infection. First, we tested the ability of RFB14KH to prevent initial *Mtb* infection in BALB/c mice when administered as pre-exposure prophylaxis (Fig. 6a). For this, BALB/c mice were treated with a single subcutaneous injection (50 μL) of placebo (*n* = 6) or RFB14KH (*n* = 6). Two weeks later, both groups of mice were exposed to the virulent Erdman strain of *Mtb* via aerosol delivery. At this time, four additional untreated mice were also exposed to aerosolized *Mtb* to determine the absolute dose of *Mtb* delivered to the lung. By homogenizing and plating out the entirety of the lung (~0.2 g of tissue) of these four animals 1-day post-aerosol exposure, the infectious dose delivered was determined to be 185 ± 15 CFU *Mtb* (or 1013 ± 158 CFU per gram of lung tissue) (Supplementary Fig. 5a). Four weeks after *Mtb* exposure, placebo and RFB14KH treated mice were necropsied, and the lung, liver, and spleen were analyzed for bacterial burden. Placebo-treated mice exhibited a more than 3-log increase in bacterial burden in the lung over time (1.8 × 10^6^ ± 4.4 × 10^5^ CFU g^−1^, mean ± S.E.M.) and substantial dissemination to distal organs including liver (4.2 × 10^4^ ± 3.6 × 10^4^ CFU g^−1^, mean ± S.E.M.) and spleen (2.5 × 10^5^ ± 1.7 × 10^5^ CFU g^−1^, mean ± S.E.M.) (Fig. 6b). Individual colony counts for each dilution and the weight of each organ homogenized is presented in Supplementary Table 2. The lungs of placebo-treated mice also exhibited gross pathological changes characterized by altered lung structures, immune infiltrates, thickened alveolar walls, and disorganized granulomatous lesions that are consistent with *Mtb* infection and typical of mice infected with *Mtb* (Fig. 6c)^47^. In contrast, RFB14KH treated mice had no detectible bacterial burden or any pathology associated with *Mtb* infection in any organ analyzed (Fig. 6b, c). Further, lungs from mice that received RFB14KH and were *Mtb* infected were not visibly different from uninfected mice that also received RFB14KH (Supplementary Fig. 5b). To alleviate concerns of drug carryover from the tissues, we concurrently plated organ homogenate on plates containing activated charcoal to precipitate any remaining drug. Importantly, there was still no growth from any drug-treated tissue (Supplementary Fig. 5c). However, the presence of charcoal negatively impacted bacterial growth, and placebo-treated tissues exhibited reduced colony growth on plates containing charcoal. These results show that pre-exposure prophylaxis with a single subcutaneous injection of RFB14KH efficiently prevents *Mtb* infection and the development of its associated pathology.

Having established the ability of RFB14KH to prevent infection, we also used BALB/c mice to assess its ability to control acute *Mtb* infection (Fig. 6d). For this purpose, BALB/c mice (*n* = 22) were exposed to *Mtb* via aerosol. The *Mtb* dose delivered in this experiment was determined 24 h later in four mice (225 ± 21 CFU per mouse, 1229 ± 128 CFU per gram of lung tissue, Supplementary Fig. 5d). One week after *Mtb* exposure, bacterial burden in the lungs, liver, and spleen in 6 of the 22 mice was assessed. At this time, all 6 mice analyzed had detectable bacterial burden in the lungs (8.8 × 10^5^ ± 1.4 × 10^4^ CFU g^−1^, mean ± S.E.M.). At this time point there was no detectible bacterial burden in the liver or spleen (Fig. 6e). Individual colony counts from 1 week post exposure are presented in Supplementary Table 3. The remaining mice were treated with a single 50 μL subcutaneous injection of either placebo (*n* = 6) or RFB14KH (*n* = 6). Three weeks after treatment initiation (four weeks after *Mtb* exposure), placebo and RFB14KH treated mice were necropsied, and the lungs, liver, and spleen were analyzed for bacterial burden and pathological manifestations of *Mtb* infection. Placebo-treated mice had *Mtb* in all organs analyzed (1.7 × 10^6^ ± 3.2 × 10^5^ CFU g^−1^ lung; 1.5 × 10^4^ ± 9.7 × 10^3^ CFU g^−1^ liver; 2.6 × 10^5^ ± 7.2 × 10^4^ CFU g^−1^ spleen, mean ± S.E.M., Fig. 6f). Individual colony counts are presented in Supplementary Table 4. Pathological changes consistent with *Mtb* disease including granulomatous lesions were observed on stained lung sections from all placebo-treated mice (Fig. 6g). In contrast, no *Mtb* was detected in mice treated with RFB14KH and no pathological changes in tissues were noted (Fig. 6f, g). Therefore, a single injection of RFB14KH administered 1 week after infection was able to efficiently reduce bacterial burden in the lung and to prevent *Mtb* dissemination to distal organs in mice exposed to *Mtb*.

## Discussion

The use of LA drug formulations is a promising strategy that can potentially improve treatment adherence; however, further evaluation of LA formulations in clinical settings is required to assess adherence^8,9^. The most important factor in the development of effective LA formulations is the daily drug release (drug release rate) that leads to sufficient plasma concentration to kill or inhibit *Mtb* in vivo.

ISFI technology allows for extensive optimization of release rates by changes in formulation composition. Indeed, statistical analysis showed higher release rates in formulations with low MW polymer (i.e., 10.6 kDa) compared to formulations with high MW polymer (i.e., 22.9 and 36.8 kDa) (Supplementary Fig. 1c) and in formulations containing DMSO compared to those containing NMP as solvent (Supplementary Fig. 1d). However, formulations with high daily release rates also require high drug load to maintain sustained drug release for long periods of time. Therefore, a high drug load in the formulation is an additional important factor for LA formulation development.

Amphiphilic additives Kolliphor^®^HS 15, TPGS, Tween 80, Tween 20, and Pluronic F68 and F127 were able to dramatically increase the solubility of RFB in DMSO and NMP (Fig. 2a and Supplementary Fig. 2) and allowed for significantly increased drug loads in RFB formulations (Fig. 2b), decreased initial release bursts (Fig. 2d) and extended drug release in vitro (Fig. 2c, e) and in vivo (Fig. 2f). Formulations with amphiphilic additives (RFB9K and RFB14K) showed faster cumulative drug release compared to RFB9 and RFB14 with the same drug load but without Kolliphor^®^HS 15. The increase was achieved mainly by an increased release burst. This is consistent with a previous report showing that the amphiphilic additive Tween 80 increased release of buprenorphine hydrochloride by increasing the initial burst with a minimal effect on later phases of drug delivery^48^. Thus, the lower release burst and extended RFB release observed in formulation RFB14KH is caused by the increase in drug load rather than the presence of amphiphilic additives. Biodegradable implants degrade by bulk degradation that depends on diffusion of water into the implant^49,50^. Degradation can be slowed by suppressing water invasion into the implant by increasing implant hydrophobicity. Therefore, implants with higher load of a hydrophobic drug are expected to have higher hydrophobicity and slower implant erosion^51^. Indeed, we showed that RFB14KH implants with a high drug load (294 mg g^−1^ RFB) had slower erosion than similar placebo implants or implants with a lower drug load (130 mg g^−1^ RFB) (Fig. 4e).

SEM surface evaluation of implants solidified from RFB14 and RFB14K that differ only by the presence of Kolliphor^®^HS 15 in RFB14K showed very similar changes in porous area and pore size during implant erosion (Fig. 5). However, at 3 days of incubation (end of implant solidification^14^), implants from the formulation containing Kolliphor^®^HS 15 (RFB14K) have a larger porous area compared to implants with the same drug load without Kolliphor^®^HS 15 (RFB14). Because the pore size at 3 days of incubation is similar between formulations, the higher porous area of RFB14K could be attributed to an increase in the number of pores present in the implant (Fig. 5a). This is consistent with the higher release burst in RFB14K, as more pores leads to increased water invasion into the implant and faster release of the drug. Interestingly, implants from RFB14KH, which had Kolliphor^®^HS 15 and a high drug load, also has higher porous area and more pores than RFB14 at 3 days of incubation, but contrary to RFB14K, it had a lower release burst. While porous area and pore size increased significantly in RFB14K throughout the implant erosion process, RFB14KH had minimal changes in porous area and pore size for the first 28 days of incubation. It is thus possible that a higher load of hydrophobic drug in the formulation resulted in protection of the implant from erosion as well as a reduced initial release burst.

Subcutaneous administration of RFB14KH (50 μL, drug load 294 mg g^−1^ RFB) to BALB/c mice resulted in plasma concentrations of RFB that were 10 times higher than the MIC for at least 4 weeks post administration and that were above the MIC for 16 weeks post administration (Fig. 3b). In fact, administration of 50 μL of RFB14KH resulted in plasma concentrations which are higher than what is expected in humans with oral dosing of 300 mg day^−1^. To avoid potential adverse effects such as uveitis and neutropenia due to high levels of RFB, the injected volume of the LA-RFB formulation in humans will have to be adjusted accordingly. Furthermore, administration of the LA-RFB formulation resulted in high penetration of drug into tissues. The mean tissue-to-plasma ratio for the lung was 16.4 and for the spleen was 20.1 (Fig. 3c). This is higher than expected. In comparison, daily oral administration of RFB (10 mg kg^−1^ day^−1^) in mice resulted in a mean tissue to plasma ratio of 3.3 for the lung and 1.74 for the spleen^52–54^. The high tissue penetration observed in our study is likely due to sustained drug delivery compared to the peak-to-trough drug concentration fluctuation in mice administered with repeated oral dosing. The high plasma concentrations and especially high tissue penetration are promising properties of the LA-RFB formulation that would allow translation to larger animals and humans. Preliminary estimates using empiric conversion based on body surface area between mice and non-human primates or humans (3.1 and 12.3, respectively^55,56^) confirm the feasibility of the use of <600 μL and <2 mL injections, respectively, that could provide adequate drug level for at least 1 month^21,22,55,57^.

The standard of care for active TB infection requires multidrug therapy^2,5^. However, a single-drug anti-TB therapy, including a 4-month rifampin regimen has been used for latent TB^58^. While clinicians sometimes substitute rifabutin for rifampin in this regimen, rifabutin has not been studied in a randomized trial for latent TB treatment^59,60^. In a model for pre-exposure treatment, RFB14KH prevented initial *Mtb* infection from occurring. Furthermore, in a model of acute *Mtb* infection, RFB14KH successfully cleared *Mtb* infection from the lung and prevented its dissemination to distal organs. Importantly, granulomatous lesions which are normally associated with *Mtb* infection in mice were not observed in any animal that received RFB14KH.

The LA-RFB formulation described herein has significant clinical potential. Our formulation based on ISFI technology has a unique composition that allows for sustained release of rifabutin. However, it includes several components that are already approved by the FDA for existing ISFI formulations including PLGA, DMSO, and Kolliphor^®^HS 15^20–24,61^. Further pre-clinical assessment is, however, required. For example, the development of a single ISFI containing multiple anti-TB drugs would be greatly beneficial for treatment of active TB. In addition, pre-clinical analysis in larger animals such as non-human primates would be beneficial to assess LA-RFB in the context of LTBI.

Taken together, we successfully developed a long-acting RFB formulation that solidifies into an implant upon subcutaneous injection and release of drug for 16 weeks. LA-RFB showed high efficacy in pre-exposure and post-exposure prophylaxis mouse models, making this approach a promising strategy for further pre-clinical development.

## Methods

### Materials

Poly (D,L-lactic-co-glycolic acid) (Lactic acid (LA):glycolic acid (GA) = 50:50, ester end group, (PLGA)) polymers with different molecular weights (MW: 36.8, 22.9, or 10.6 kDa) were purchased from LACTEL^®^ Absorbable Polymers (AL, USA). Rifabutin (RFB) and rifampin (RIF) were purchased from Cayman Chemical (MI, USA). Dimethyl sulfoxide (DMSO), acetonitrile with 0.1% formic acid, and Tween 80 were bought from Fisher Scientific (MA, USA). N-Methyl-2-pyrrolidone (NMP), ethylenediaminetetraacetic acid (EDTA), Kolliphor^®^HS 15 (Solutol), pluronic F127, F68, Tween 20, D-α-Tocopherol polyethylene glycol 1000 succinate (TPGS), phosphate-buffered saline (PBS, pH 7.4), and PLGA with acid-ending (LA:GA = 50:50, acid end group, Resomer^®^ RG 502 H, 13.5 kDa) were purchased from Sigma-Aldrich (MO, USA).

### RFB solubility in DMSO and NMP

DMSO or NMP (50 μL) was added to 15 mg of RFB. The mixed samples were incubated for 1 day at room temperature (RT) using a rotary shaker (Barnstead/Thermolyne Model 415110 Labquake Shaker/rotator, Thermo Fisher Scientific, USA). The undissolved RFB in the solvent was removed by centrifugation (21,130 × *g*, RT, 5 min) (Eppendorf Centrifuge 5417R, Eppendorf Inc., Germany) and RFB concentration in the supernatant was analyzed by measuring the absorbance at 320 nm using a UV-vis spectrometer (SpectraMax M2/M2e microplate reader, Molecular Devices, CA, USA, with DeNovix DS-11 software version v 4.1.9, Wilmington, DE, USA). All experiments were performed in triplicate.

### RFB solubility with different amphiphilic additives

The effect of different amphiphilic additives on RFB solubility was investigated using various concentrations in biocompatible solvents. Kolliphor^®^HS 15 was prepared in DMSO (concentration range: 0.045–47.6 wt%) and NMP (concentration range: 0.45–47.6 wt%). Pluronic F127, F68, Tween 80, Tween 20, and TPGS were dissolved in DMSO at concentration range: 0.0009–8.3 wt%. RFB was then mixed with the prepared solutions to a target of 900 mg mL^−1^ RFB solution, and the mixtures were incubated on a rotary shaker for 1 day at RT. The undissolved RFB in the solution was removed by centrifugation (21,130 × *g*, 5 min, RT), and the supernatant was collected. RFB concentration in the supernatant was measured by absorbance using a UV-vis spectrometer (DeNovix DS-11). All experiments were performed in triplicate.

### Preparation of LA-RFB formulations

RFB was mixed with the solvent (DMSO or NMP) with or without 0.45 wt% Kolliphor^®^HS 15 at the maximum saturated concentration and incubated in a rotary shaker for 1 day at RT. PLGA at different mass ratios to solvent (solvent:polymer = 2:1, 4:1, or 5:1) was added and incubated in the rotary shaker over 1 day at RT in the dark or until polymer dissolved. RFB9K and RFB14K formulations were prepared by dissolving RFB in DMSO with 0.45 wt% Kolliphor^®^HS 15 at concentration 202 mg mL^−1^. PLGA ester-capped (RFB9K) or acid-ending (RFB14K) was added at solvent: polymer = 4:1 mass ratio and mixture was incubated in rotary shaker for 1 day to dissolve polymer. The prepared formulations were then stored in the dark at RT. Drug load (%) was calculated according to Eq. (1)^39^:

1 $${{{{{{\rm{Drug}}}}}}}\,{{{{{{\rm{load}}}}}}}\,\left(\%\right)=\frac{{{{{{{\rm{weight}}}}}}}\,{{{{{{\rm{of}}}}}}}\,{{{{{{\rm{drug}}}}}}}}{{{{{{{\rm{weight}}}}}}}\,{{{{{{\rm{of}}}}}}}\,({{{{{{\rm{drug}}}}}}}+{{{{{{\rm{polymer}}}}}}})}\times 100$$

### In vitro release properties of LA-RFB formulations

RFB in vitro release from LA-RFB was evaluated in phosphate-buffered saline (PBS, pH 7.4), at 37 °C in an orbital shaker (100 rpm) under sink conditions (concentration of RFB in release medium <42 μg). Specifically, 30 µl of the prepared RFB-ISFI formulation was directly injected into 10 mL of PBS (pH 7.4) using a 1 mL syringe with a 19-gauge needle. At pre-determined time points (1, 2, 3, 5, 7, 10, 14 days, and once a week up to 14 weeks of incubation), 1 mL of solution was collected, and the total release buffer was replaced with fresh PBS. After 14 weeks, the remaining implants were collected and dissolved in 1 mL DMSO. The RFB concentration in the collected release medium and the dissolved implant samples were measured using a UV-vis spectrometer (SpectraMax M2/M2e microplate reader, Molecular devices, CA, USA with DeNovix DS-11 software, Wilmington, DE, USA). All experiments were performed in triplicate. RFB release was evaluated as cumulative release of RFB in μg or percentage of injected RFB^62,63^ (Eq. 2). The release rate of RFB^64^ at each sampling time point were calculated according to Eq. (3) using Microsoft Excel (2021, version 16.57):

2 $${{{{{\rm{Cumulative}}}}}}\; {{{{{\rm{RFB}}}}}}\; {{{{{\rm{percentage}}}}}}\left(\%\right)=\frac{{V}_{0}{\sum }_{i=1}^{n}{{{{{{\rm{C}}}}}}}_{i}}{{m}_{0}}\times 100$$

3 $${{{{{\rm{RFB}}}}}}\; {{{{{\rm{release}}}}}}\; {{{{{\rm{rate}}}}}}\,({{\upmu }}{{{{{\rm{g}}}}}}\; {{{{{\rm{da}}}}}}{{{{{{\rm{y}}}}}}}^{-1})=\frac{{V}_{0}({{{{{{\rm{C}}}}}}}_{{{{{{\rm{n}}}}}}}-{{{{{{\rm{C}}}}}}}_{{{{{{\rm{n}}}}}}-1})}{{t}_{{{{{{\rm{n}}}}}}}-{t}_{{{{{{\rm{n}}}}}}-1}}$$

Where *V*_0_ is the total volume of release media, C_*i*_ is the RFB concentration (mg mL^−1^) in the release solution, m_0_ is the weight of RFB in the formulations injected to release medium, and *t*_n_ is time (days) of sample collected, C_n_ is the RFB concentration in release media at day of collection. Initial release burst was defined as cumulative percentage of total RFB released within the first 72 h of incubation in release medium.

### In vivo pharmacokinetics of LA-RFB formulations

All animal protocols were approved by the Institutional Use and Care Committee at the University of North Carolina-Chapel Hill and with reference to the National Institutes of Health Guide for the Care and Use of Laboratory Animals, approval number 21-134. BALB/c mice (females, 10–12 weeks old, *n* = 3–5, The Jackson Laboratory), were housed in SPF animal facility with ambient temperature (20–23 °C), 30–70% humidity, and 12 h dark/light cycle. Mice were administered subcutaneously with 50 µL of the LA-RFB formulation using a 19-gauge needle. Mice were bled at pre-determined time points (3, 7, 14, 21, 28, 35, and 42 days after injection, then biweekly) until plasma RFB concentrations decreased below the MIC. Peripheral blood was collected in EDTA-coated tubes and plasma isolated by centrifugation (5 min, 300 g) and stored at −80 °C until analysis by HPLC.

### HPLC analysis of plasma rifabutin concentrations

Plasma RFB concentrations were measured by HPLC analysis using rifampin (RIF) as an internal standard^65^. Briefly, 36 µl of plasma was mixed with 4 µl of 100 µg mL^−1^ RIF solution in DMSO followed by the addition of 60 µL of acetonitrile with 0.1% formic acid. Excess protein was removed by centrifugation (5 min, 13,523 × *g*, 4 °C) (Eppendorf 5424 Microcentrifuges, Eppendorf Inc., Germany). Supernatant (80 µl) was then collected and dried for 1 h using a vacuum centrifuge (Eppendorf 5301 Vacufuge Concentrator Centrifuge, Eppendorf Inc., Germany). The dried sample was resuspended in 32 µL of acetonitrile with 0.1% formic acid. The resuspended samples were centrifuged (5 min, 13,523 × *g*, 4 °C) and supernatants collected. RFB concentration in supernatant was measured using HPLC (Agilent Technologies 1200 series HPLC system with Agilent ChemStation Software version B.04.01 LC 1200 (Agilent Scientific Instruments, USA). HPLC condition: 40 °C, running time = 10 min, C18 column with a mobile phase composed of co-solvent (acetonitrile:water = 60:40) at a rate of 1 mL min^−1^; detection wavelength: 275 nm).

### Analysis of RFB in tissues

RFB in mouse tissue was extracted from calibration standards, quality control samples, and study samples using protein precipitation and LC-MS/MS analysis. Tissue samples were initially homogenized in phosphate-buffered saline (PBS) with 100 μg mL^−1^ cycloheximide and 50 μg mL^−1^ carbenicillin. The resulting homogenate was mixed with methanol in a 1:3 ratio. Fifty microliters of resulting sample was extracted by protein precipitation with methanol containing RFB-d_7_ (RFB-IS) as an internal standard. Following vortex and centrifugation, a portion of the supernatant was diluted with water 1:1 prior to LC-MS/MS analysis. RFB was eluted from a Phenomenex Synergi Polar-RP (50 × 2.0 mm, 2.5 μm particle size) analytical column. Data were collected using Sciex Analyst Chromatography Software on an API-5000 triple quadruple mass spectrometer (version 1.7.1, SCIEX, Foster City, CA, USA). Calibration curves were obtained by using a 1 concentration^−2^ weighted linear regression of analyte:internal standard peak area ratio vs. concentration. The calibration curve for this assay was 1–20,000 ng mL^−1^ homogenate. All calibrators and quality control samples were within 15% of the nominal concentrations.

### Stability of LA-RFB formulations

RFB14 and RFB14KH were stored at room temperature in the dark. At pre-determined time points (3, 4, 6, 9, 12, 15, and 18 months of incubation) 10 μL of formulation was diluted in 1 mL of DMSO, then further diluted at 1:1000 in DMSO for analysis by UV-vis absorbance spectrometer and HPLC to measure the drug concentration and drug degradation. As a control, RFB dissolved in DMSO (20 μg mL^−1^ RFB solution) was incubated in the dark at RT.

### Scanning electron microscopy (SEM) imaging of LA-RFB implants

Implants were prepared by injection of 30 µL LA-RFB formulation into 10 mL of PBS (pH 7.4) and incubated at 37 °C with 100 rpm shaking. The PBS buffer was replaced with fresh PBS following the same time schedule as the in vitro release experiment. At pre-determined time points (3, 7, and 28 days after incubation), the implants were collected and lyophilized for 24 h. The lyophilized implants were fractured by razor on dry ice to investigate their internal structure. Implants were mounted on an aluminum platform using carbon tape. The mounted implants were coated with 5 nm of gold-palladium alloy (60:40) (Hummer X Sputter Coater, Anatech USA, Union City, CA). The coated samples were imaged using a Zeiss Supra 25 field emission scanning electron microscope with an acceleration voltage of 5 kV, 30 m aperture, and average working distance of 10 mm and Zeiss ZEN 2011 software (version 1.0, Carl Zeiss Microscopy, LLC, Thornwood, NY)^14,66^. Imaging was completed in a blinded manner. Pore size and porous area on the surface of implants were measured from the SEM images using ImageJ software (ImageJ 1.53k, Java 1.8.0_172 [64-bit], NIH, Maryland)^67^. The porous area was calculated as a percentage of pore area in total area of the scanned SEM image. The pore size and porous area on the surface implants were measured on three regions of each implant, one to three different implants were analyzed.

### *Mtb* infection and LA-RFB efficacy in vivo

All studies involving *Mtb* were carried out under Biosafety Level 3 (BSL-3) containment. BALB/c mice (females, 12–14 weeks old, The Jackson Laboratory), were transferred to the BSL-3 facility and allowed to rest for one week prior to experimentation. The animal protocol for the study was approved by the Institutional Use and Care Committee at the University of North Carolina-Chapel Hill and with reference to the National Institutes of Health Guide for the Care and Use of Laboratory Animals, approval number 21-168. Mice were housed in ambient temperature (20–23 °C), 30–70% humidity, and 12 h dark/light cycle.

*Mtb* Erdman was grown at 37 °C in liquid Middlebrook 7H9 medium supplemented with 0.05% Tween 80, 0.5% glycerol, and 1x albumin-dextrose-saline (>0.5% bovine serum albumin, 0.2% glucose, 0.85% NaCl). Mice were placed in a Madison aerosol chamber (Mechanical Engineering Workshop, Madison, WI) calibrated to deliver ~250 CFU *Mtb*. After each exposure, four mice were sacrificed one day post infection to determine bacterial uptake. Whole lungs were weighed and then homogenized in 5 mL PBS supplemented with 100 μg mL^−1^ cycloheximide, and 50 μg mL^−1^ carbenicillin. Diluted homogenate was then plated on 7H10 plates supplemented with 0.5% glycerol, 10% OADC (oleic acid, bovine albumin, dextrose, and catalase), 100 μg mL^−1^ cycloheximide, 50 μg mL^−1^ carbenicillin, and 15 μg mL^−1^ trimethoprim for CFU enumeration. Charcoal plates were supplemented with 0.4% activated charcoal ^68^.

Either 2 weeks prior to exposure or 1 week after exposure, mice received a single subcutaneous injection of 50 μL LA-RFB or placebo using a 1 mL syringe and 19-gauge needle. For the prevention study, researchers assessing bacterial burden were blinded to treatment group. Groups of mice were sacrificed at 28 days post-infection, and lungs, livers, and spleens collected for analysis. A small piece of tissue was retained for histology in 10% formalin over 24 h prior to removal from the BSL-3. The remaining tissue was weighed and processed for CFU enumeration. Bacterial burden is presented as CFU g^−1^ instead of CFU per organ because a portion of the tissue was retained for histology, so the whole tissue was not homogenized. Each tissue was homogenized in 5 mL PBS supplemented with 100 μg mL^−1^ cycloheximide, and 50 μg mL^−1^ carbenicillin. For each organ, undiluted, 1:10, 1:100, and 1:1000 serial dilutions were plated for CFU enumeration. Initially, we utilized quadrant plates, wherein 28 μL of organ homogenate was plated. However, full plates (100 mm × 15 mm) and a larger plating volume (111 μL) were used in later studies in order to lower the limit of detection. Drug carryover was reduced using agar supplemented with 0.4% activated charcoal (Supplementary Fig. 5c) and by a dilution protocol that effectively lowered the amount of RFB plated, so drug carryover did not interfere with CFU enumeration in LA-RFB treated animals. Specifically, in the lung, liver, and spleen, we observed less than 10 μg RFB per gram of tissue (mean lung RFB was 6.68 μg at 6 weeks post injection, Fig. 3c). Each lung weighed ~0.2 g. RFB carryover with 111 μL plated on a 10 cm plate with 30 mL agar was 30 ng for undiluted samples, 3 ng, 0.3 ng, and 0.03 ng for a 1:10, 1:100, and 1:1000 dilution, respectively.

Fixed tissue was removed from the BSL-3, paraffin embedded, and cut into 5-μm sections, which were mounted onto Superfrost Plus slides (Fisher Scientific). Tissue sections were incubated at 60 °C for one hour, followed by deparaffinization with xylene (2 × 3 min) and rehydration in graded ethanol (100% 2 × 3 min, 95% 1 × 3 min, 80% 1 × 3 min, 70% 1 × 3 min) and ddH_2_O (10 min). Sections were stained with hematoxylin for 15 min, washed in water for 20 min, and stained with eosin for one min. Following ethanol and xylene incubation (95% ethanol 2 × 2 min, xylene 2 × 2 min), slides were mounted and imaged on a Nikon Eclipse Ci microscope using Nikon Elements BR software (version 4.30.01) with a Nikon Digital Sight DS-Fi camera. ImageJ/Fiji (version 2.1.0/1.53f) was used to adjust brightness and contrast on whole images. Although images were not taken in a blinded manner due to apparent differences in tissue pathology between treated and untreated groups, images were obtained and evaluated from all mice in all treatment and placebo groups.

### Statistical analysis

No statistical methods were used to predetermine sample size. All statistical analyses were completed using GraphPad Prism (version 9.0.0). Differences in drug load between formulations with and without additives were analyzed using a Mann–Whitney U test (Fig. 1d). Differences in release rates from LA-RFB formulations based on polymer MW were determined using a one-way ANOVA for each solvent to polymer ratio (Supplementary Fig. 1c). Correction for multiple comparisons was controlled using the False Discovery Rate and the two-stage step-up method of Benjamini, Krieger, and Yekutieli. Similarly, a one-way ANOVA followed by correction for multiple comparisons was used to compare all formulations with a low MW PLGA (Supplementary Fig. 1d). Differences in RFB drug load and initial release burst were compared using a unpaired *t*-test (Fig. 2b, d) Differences in the RFB tissue concentration and the tissue to plasma ratio at 2 and 6 weeks post treatment were analyzed using a Mann–Whitney U test for each organ (Fig. 3c, d). Differences in the porous area and pore size for each implant over time (Fig. 5b, c) were analyzed using a one-way ANOVA for each formulation with multiple comparisons as in Supplementary Fig. 1c. To determine the difference between CFU burden in treated and untreated animals (Fig. 6b, g), a Mann–Whitney U test was completed for each organ.

### Reporting summary

Further information on research design is available in the Nature Research Reporting Summary linked to this article.

## Supplementary information

Supplementary Material

Reporting Summary

## Data Availability

The authors declare that all data supporting the findings of this study are available within the paper and its supplementary information files. Source data are provided with this paper.

## Source data

Source Data
