# Supplementary material for: A long-acting formulation of rifabutin is effective for prevention and treatment of Mycobacterium tuberculosis
Source: Nat Commun. 2022 Aug 8;13:4455. doi: 10.1038/s41467-022-32043-3 (PMC9360445; doi:10.1038/s41467-022-32043-3)
Supplement: Supplementary file 1 — Supplementary Material [file 41467_2022_32043_MOESM1_ESM.pdf]

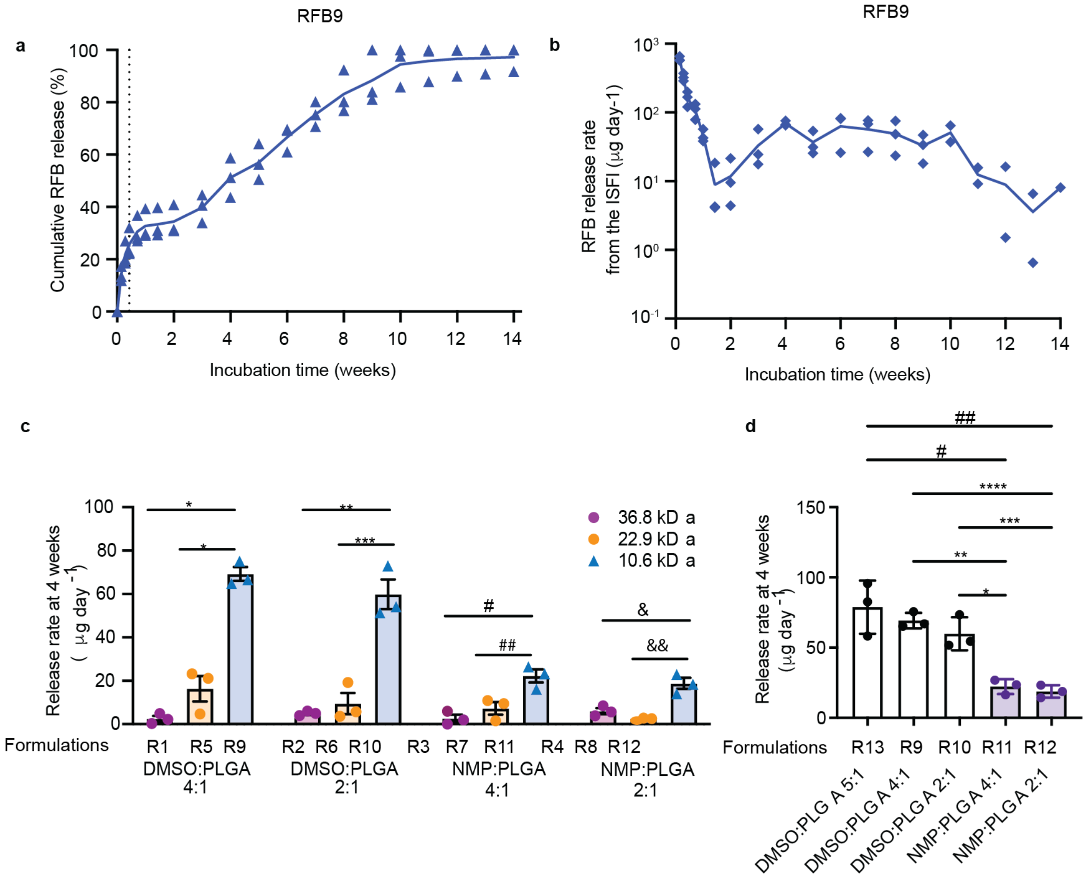

Supplementary Material

Supplementary Fig. 1: In vitro release properties of LA-RFB depend on polymer and solvent composition. LA-RFB formulations were evaluated under sink conditions after injecting 30 µL of the formulation into release medium (PBS). **a** Cumulative release of RFB from the formulation (RFB9) as a percent of the initially injected RFB. Dotted line indicates initial burst, the cumulative RFB released within the first 72 h post injection, mean and individual replicates are shown. **b** RFB daily release (µg day^{-1}) over time, mean and individual replicates are shown. **c** RFB daily release (ug day^{-1}) from LA-RFB formulations with polymers of different MW and indicated solvent: polymer ratios four weeks post injection. R1-12 indicate formulations RFB1-12 (Table 1), *P=0.0001, **P=0.0005, ***P=0.0008, $^{\#}$P=0.0045, $^{\#\#}$P=0.0164, $^{\&}$P=0.004, $^{\&\&}$P=0.0012. **d** RFB daily release from LA-RFB with 10.6 kDa PLGA and different solvents at four weeks of incubation. R1-12 indicates formulations RFB1-12 (Table 1), *P= 0.011,**P=0.0023, ***P=0.006, ****P=0.0014, $^{\#}$P=0.0005, $^{\#\#}$P=0.0007. For panels **a-d** n=3, in **a,b** means and individual values are shown, in **c,d** means ± SD are shown. Statistical significance in panel c and d was determined using a one-way ANOVA.

Supplementary Table 1. In vitro properties of LA-RFB formulations with Kolliphor®HS 15 and acid-ending PLGA

Formulation	PLGA MW [kDa]	Solvent	Solvent: polymer ratio	Drug load [mg g^{-1}]	Injectable [a]	Initial release burst [%][b]	Release rate at 4 weeks [µg day^{-1}]	Release rate at 8 weeks [µg day^{-1}]	Release rate at 12 weeks [µg day^{-1}]
RFB9KH	10.6	DMSO with 0.45 wt% Solutol	4:1	293	Y	7.4 ± 0.2	48.3 ± 16.2	34.5 ± 4.5	10.4 ± 2.6
RFB11KH	10.6	NMP with 8.8% Solutol	4:1	352	Y	9.0 ± 1.3	121.4 ± 35.7	44.5± 3.4	31.8± 15.2
RFB13KH	10.6	DMSO with 0.45 wt% Solutol	5:1	297	Y	8.1 ± 0.5	53.3 ± 2.4	23.4 ± 9.3	15.4 ± 8.4
RFB9K [c]	10.6	DMSO with 0.45 wt% Solutol	4:1	128	Y	34.4 ± 4.5	20.7 ± 4.6	30.3 ± 15.5	N/A
RFB14	13.5 (Acid-ending)	DMSO	4:1	130	Y	12.6 ± 0.9	47.5 ± 9.0	37.0 ± 4.8	24.5 ± 5.2
RFB14KH	13.5 (Acid-ending)	DMSO with 0.45 wt% Solutol	4:1	294	Y	9.6 ± 1.6	92.6 ± 8.7	77.7 ± 13.2	67.2 ± 5.7
RFB14K	13.5 (Acid-ending)	DMSO with 0.45 wt% Solutol	4:1	132	Y	18.8 ± 5.7	27.0 ± 3.6	59.0 ± 10.2	9.7 ± 16.8

[a] Y= Injectable

[b] Initial release burst is the cumulative percentage of RFB released into the incubation medium (PBS) 72 h after the injection

[c] RFB9K implant was degraded after 9 weeks incubation in the release medium

Data are expressed by mean ± SD

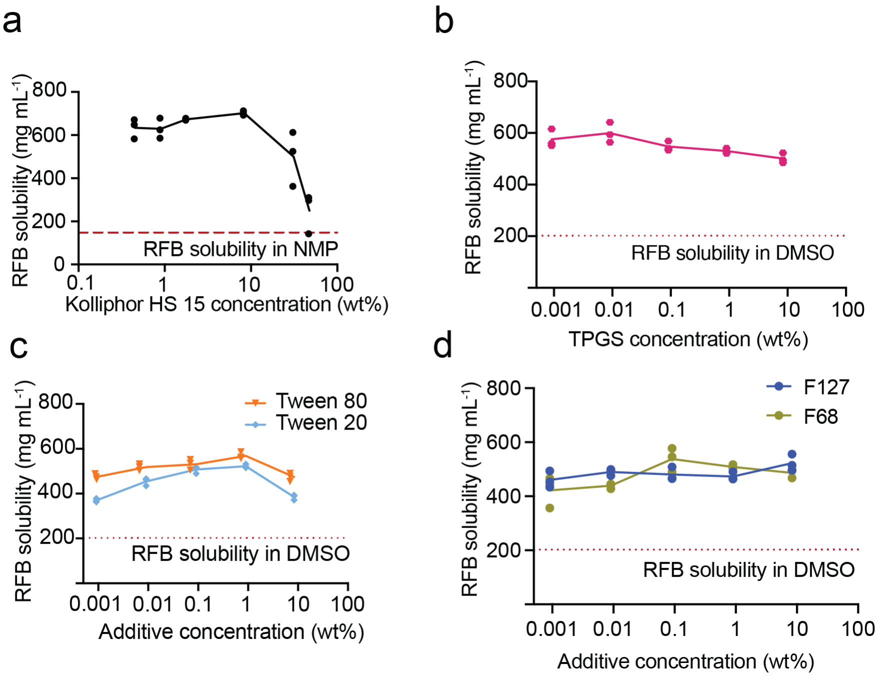

Supplementary Fig. 2: Amphiphilic additives increase the saturated solubility of RFB in organic biocompatible solvents. a The saturated solubility of RFB in NMP in the presence of indicated concentrations of Kolliphor®HS 15. **b-d** The saturated solubility of RFB in DMSO in presence of following additives: TPGS (**b**), Tween 80 or Tween 20 (**c**), and Pluronic F127 or Pluronic F68 (**d**). (n=3 per concentration, means and individual measurements are shown.) Dashed line indicates the solubility of RFB in solvent without additives.

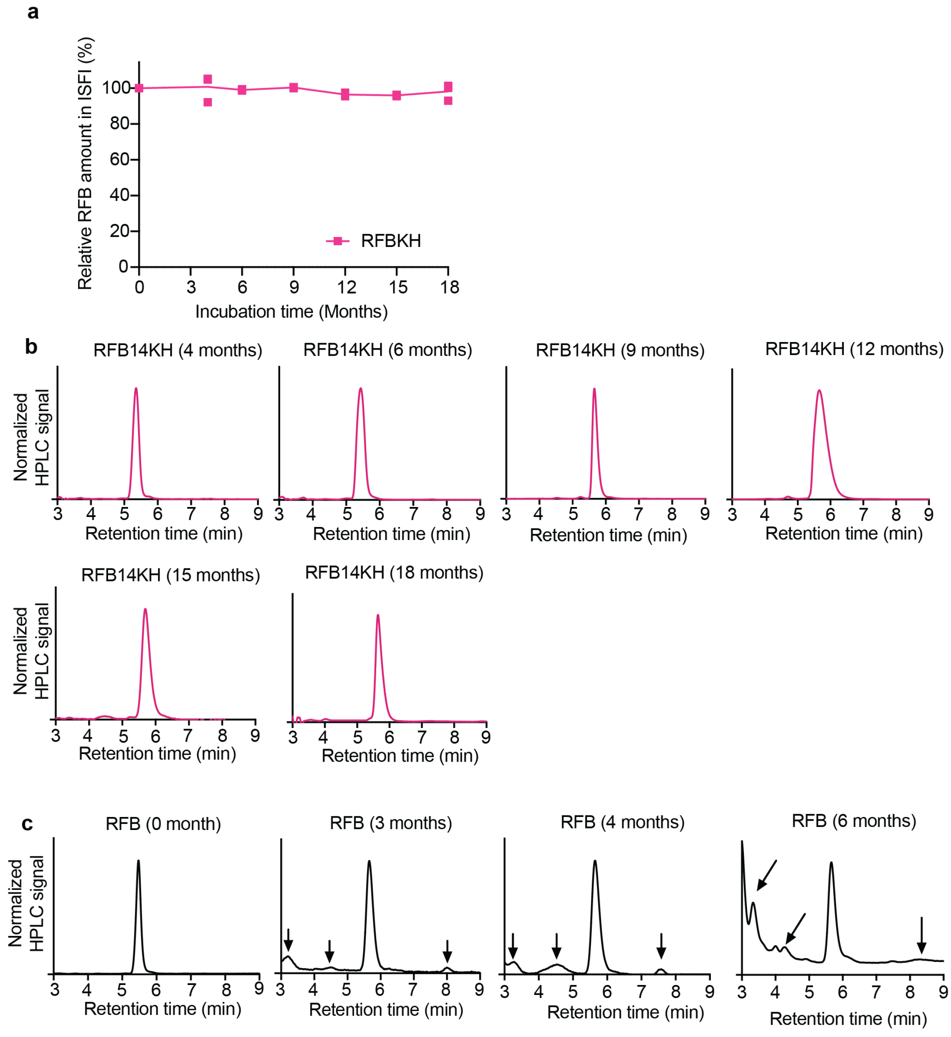

Supplementary Fig. 3: RFB stability in RFB14KH formulation. RFB14KH was stored at room temperature (25°C) in the dark for 18 months. **a** Relative RFB amounts in the formulation over time (n=3). **b,c** Normalized HPLC histograms of RFB in RFB14KH formulation, representative histograms from 3 measurements are shown (**b**) or RFB in DMSO without polymer (**c**) for 6 months; n=3; representative histograms from 3 measurements are shown

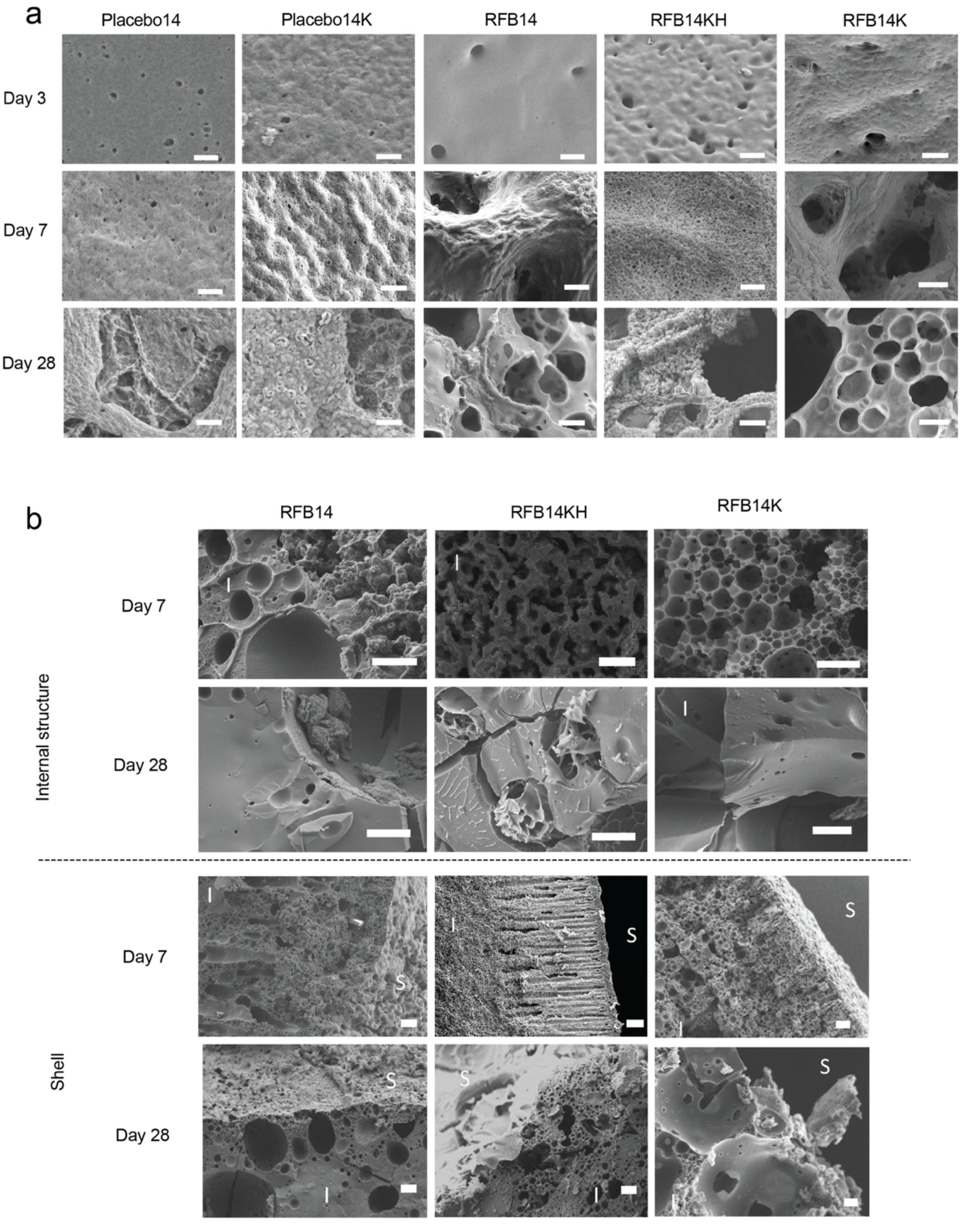

Supplementary Fig. 4: Structure of LA-RFB implants. a,b SEM images of the surface (**a**, scale bar=2 µm,) and the internal structures (**b**, scale bar=10 µm) of the implants formed by RFB14, RFB14KH, RFB14K, and the corresponding placebos (placebo14 and placebo14K) over time (for the surface: 3, 7, and 28 days of incubation, for the internal structure: 7 and 28 days of incubation) (I=inside area of implant and S=shell of implant). Representative images are shown in a) and b), three regions of each implant was scanned, three different implants were analyzed for each timepoint and formulation.

Supplementary Table 2. Colony counts and dilutions for the *Mtb* prevention experiment plated with or without charcoal

Treatment	Mouse	Tissue	Weight (g)	Undiluted		1:10		1:100		1:1000	
					+AC		+AC		+AC		+AC
Placebo	P1	lung	0.21	TMTC	TMTC	98, 106	TMTC	5, 16	7, 8	0, 2	NG
		liver	0.83	4, 7	11, 17	0, 2	NG	NG	NG	NG	NG
		spleen	0.19	TMTC	17, 4	8, 6	NG	NG	NG	NG	NG
	P2	lung	0.20	TMTC	TMTC	49, 42	25, 31	NG	NG	NG	NG
		liver	0.91	7, 5	63, 64	NG	NG	NG	NG	NG	NG
		spleen	0.07	25, 16	NG	NG	NG	NG	NG	NG	NG
	P3	lung	0.25	TMTC	TMTC	TMTC	TMTC	25, 23	12, 12	2,0	NG
		liver	0.74	23, 22	18, 28	1, 1	NG	NG	NG	NG	NG
		spleen	0.13	55, 67	11, 10	4, 10	NG	1, 1	NG	NG	NG
	P4	lung	0.23	TMTC	TMTC	TMTC	TMTC	18, 29	17, 12	1, 3	NG
		liver	1.09	TMTC	TMTC	11,16	7, 6	NG	NG	NG	NG
		spleen	0.18	TMTC	44, 30	15, 7	NG	NG	NG	NG	NG
	P5	lung	0.19	TMTC	TMTC	35, 38	44, 31	2, 5	NG	NG	NG
		liver	1.11	21, 28	63, 64	6, 7	NG	NG	NG	NG	NG
		spleen	0.19	55, 67	6, 10	4, 10	NG	1, 1	NG	NG	NG
	P6	lung	0.19	TMTC	TMTC	TMTC	50, 60	25	5, 10	1	NG
		liver	0.69	75, 74	76, 70	4, 4	5, 6	1, 0	NG	NG	NG
		spleen	0.18	TMTC	58, 55	16, 15	1, 0	1, 1	NG	NG	NG
RFB	R1	lung	0.11	NG	NG	NG	NG	NG	NG	NG	NG
		liver	0.92	NG	NG	NG	NG	NG	NG	NG	NG
		spleen	0.11	NG	NG	NG	NG	NG	NG	NG	NG
	R2	lung	0.14	NG	NG	NG	NG	NG	NG	NG	NG
		liver	0.93	NG	NG	NG	NG	NG	NG	NG	NG
		spleen	0.09	NG	NG	NG	NG	NG	NG	NG	NG
	R3	lung	0.10	NG	NG	NG	NG	NG	NG	NG	NG
		liver	0.51	NG	NG	NG	NG	NG	NG	NG	NG
		spleen	0.1	NG	NG	NG	NG	NG	NG	NG	NG
	R4	lung	0.29	NG	NG	NG	NG	NG	NG	NG	NG
		liver	0.92	NG	NG	NG	NG	NG	NG	NG	NG
		spleen	0.19	NG	NG	NG	NG	NG	NG	NG	NG
	R5	lung	0.19	NG	NG	NG	NG	NG	NG	NG	NG
		liver	0.82	NG	NG	NG	NG	NG	NG	NG	NG
		spleen	0.12	NG	NG	NG	NG	NG	NG	NG	NG
	R6	lung	0.16	NG	NG	NG	NG	NG	NG	NG	NG
		liver	0.69	NG	NG	NG	NG	NG	NG	NG	NG
		spleen	0.13	NG	NG	NG	NG	NG	NG	NG	NG

AC = activated charcoal; NG = no growth; TMTC = too many to count; dilutions were plated in duplicate on quadrant plates

Supplemental Figure 5

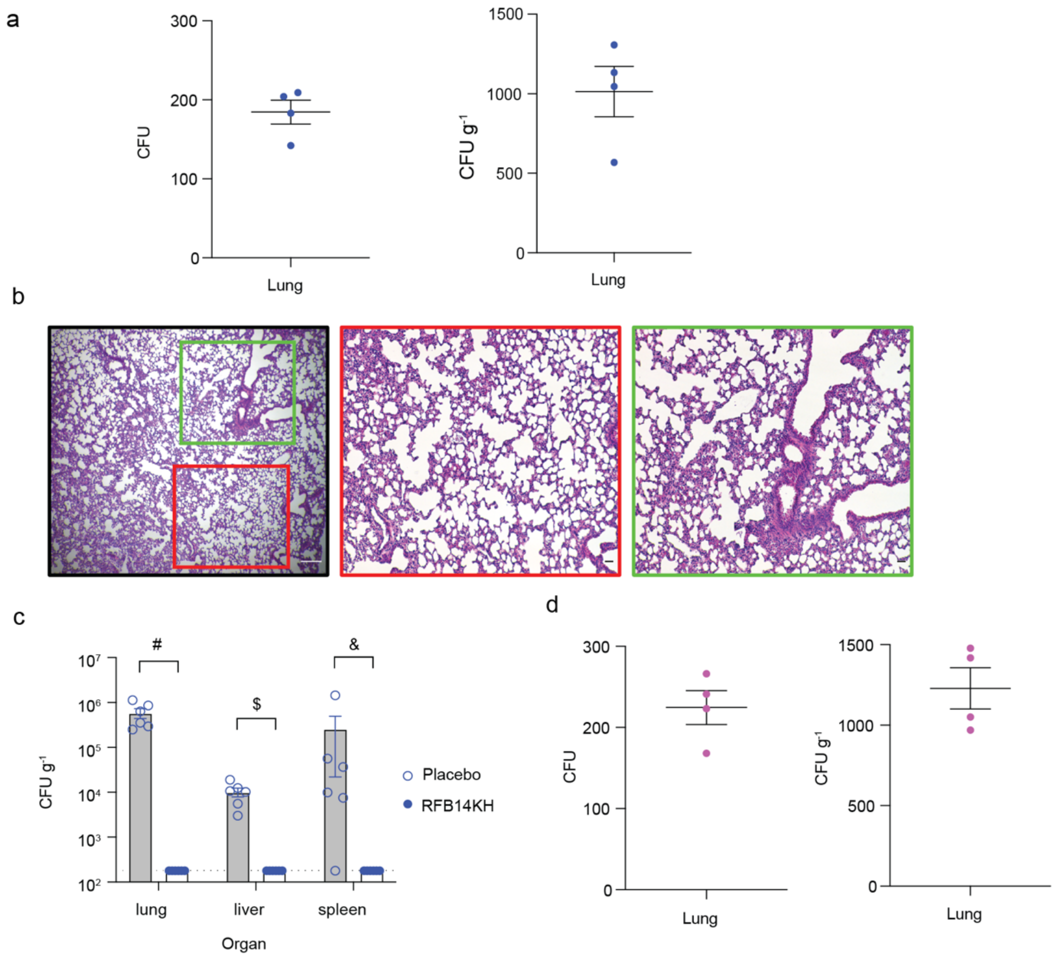

Supplementary Fig. 5: In vivo *Mtb* exposure dose and LA-RFB treated lungs. a Exposure dose for the pre-exposure prophylaxis experiment. Dose is displayed as CFU (left) and CFU g^{-1} of lung tissue (right), n=4. Data is expressed as mean ± S.E.M. **b** Representative H&E images of the lung of an uninfected BALB/c mouse treated with 50 µL RFB14KH. Mice were necropsied 4 weeks after treatment, n=4. Scale bars in low magnification images are 300 µm and 50 µm in higher magnification images. **c** Bacterial burden in the lung, liver, and spleen four weeks post infection in mice that were treated one week pre-exposure with either LA-RFB or placebo (n=6 per group). Colonies were counted from plates containing activated charcoal, (#P=0.0021, $0.0021, &P=0.0151, Mann-Whitney U test) **d** Exposure dose for the post-exposure treatment experiment. Dose is expressed as CFU (left) and CFU g^{-1} of lung tissue (right), n=4. Data in panels a, c, and d are expressed as mean ± S.E.M.

Supplementary Table 3. Colony counts and dilutions for untreated mice one week after *Mtb* infection

Treatment	Mouse	Tissue	Weight (g)	Undiluted	1:10	1:100
Untreated Week 1	U1	lung	0.10	58, 61	6, 5	1, 1
		liver	0.97	NG	NG	NG
		spleen	0.08	NG	NG	NG
	U2	lung	0.13	62, 46	3, 12	1, 0
		liver	1.19	NG	NG	NG
		spleen	0.12	NG	NG	NG
	U3	lung	0.15	43, 44	3, 15	1, 0
		liver	0.51	NG	NG	NG
		spleen	0.06	NG	NG	NG
	U4	lung	0.12	59, 70	5, 5	NG
		liver	0.92	NG	NG	NG
		spleen	0.05	NG	NG	NG
	U5	lung	0.08	67, 63	6, 1	NG
		liver	1.06	NG	NG	NG
		spleen	0.06	NG	NG	NG
	U6	lung	0.21	67, 61	11, 1	NG
		liver	1.24	NG	NG	NG
		spleen	0.06	NG	NG	NG

NG = no growth; each dilution was plated in duplicate on quadrant plates

Supplementary Table 4. Colony counts and dilutions for the *Mtb* treatment efficacy experiment

Treatment	Mouse	Tissue	Weight (g)	Undiluted	1:10	1:100	1:1000
Placebo	P7	lung	0.19	TMTC	51, 60	4, 0	1, 0
		liver	0.78	4, 0	1, 0	NG	NG
		spleen	0.09	TMTC	11, 10	0, 1	NG
	P8	lung	0.18	TMTC	TMTC	35, 24	6, 4
		liver	0.73	TMTC	19, 32	3, 5	1, 0
		spleen	0.14	TMTC	49, 41	4, 2	1, 0
	P9	lung	0.23	TMTC	TMTC	22, 22	3, 1
		liver	0.94	TMTC	12, 9	3, 0	NG
		spleen	0.11	TMTC	19, 24	1, 0	NG
	P10	lung	0.24	TMTC	TMTC	15, 24	0, 4
		liver	1.23	30, 31	4, 3	NG	NG
		spleen	0.07	TMTC	7, 11	NG	NG
	P11	lung	0.23	TMTC	TMTC	28, 14	1, 4
		liver	1.12	5, 8	0, 1	NG	NG
		spleen	0.08	45, 41	3, 3	NG	NG
	P12	lung	0.39	TMTC	TMTC	51, 36	5, 7
		liver	1.18	21, 27	5, 4	NG	NG
		spleen	0.11	TMTC	8, 7	0, 1	NG
RFB	R7	lung	0.17	NG	NG	NG	NG
		liver	0.99	NG	NG	NG	NG
		spleen	0.09	NG	NG	NG	NG
	R8	lung	0.10	NG	NG	NG	NG
		liver	0.99	NG	NG	NG	NG
		spleen	0.09	NG	NG	NG	NG
	R9	lung	0.15	NG	NG	NG	NG
		liver	0.49	NG	NG	NG	NG
		spleen	0.06	NG	NG	NG	NG
	R10	lung	0.15	NG	NG	NG	NG
		liver	0.49	NG	NG	NG	NG
		spleen	0.06	NG	NG	NG	NG
	R11	lung	0.12	NG	NG	NG	NG
		liver	0.79	NG	NG	NG	NG
		spleen	0.11	NG	NG	NG	NG
	R12	lung	0.16	NG	NG	NG	NG
		liver	0.84	NG	NG	NG	NG
		spleen	0.09	NG	NG	NG	NG

NG = no growth; TMTC = too many to count; each dilution was plated in duplicate on 100 mm plates
