# Supplementary material for: A long-acting formulation of rifabutin is effective for prevention and treatment of Mycobacterium tuberculosis
Source: Nat Commun. 2022 Aug 8;13:4455. doi: 10.1038/s41467-022-32043-3 (PMC9360445; doi:10.1038/s41467-022-32043-3)
Supplement: Supplementary file 2 — Reporting Summary [file 41467_2022_32043_MOESM2_ESM.pdf]

nature portfolio

Corresponding author(s): Martina Kovarova, J. Victor Garcia

Last updated by author(s): Jun 22, 2022

Reporting Summary

Nature Portfolio wishes to improve the reproducibility of the work that we publish. This form provides structure for consistency and transparency in reporting. For further information on Nature Portfolio policies, see our Editorial Policies and the Editorial Policy Checklist.

Statistics

For all statistical analyses, confirm that the following items are present in the figure legend, table legend, main text, or Methods section.

n/a	Confirmed	
☐	☒	The exact sample size (*n*) for each experimental group/condition, given as a discrete number and unit of measurement
☐	☒	A statement on whether measurements were taken from distinct samples or whether the same sample was measured repeatedly
☐	☒	The statistical test(s) used AND whether they are one- or two-sided<br>*Only common tests should be described solely by name; describe more complex techniques in the Methods section.*
☒	☐	A description of all covariates tested
☐	☒	A description of any assumptions or corrections, such as tests of normality and adjustment for multiple comparisons
☐	☒	A full description of the statistical parameters including central tendency (e.g. means) or other basic estimates (e.g. regression coefficient) AND variation (e.g. standard deviation) or associated estimates of uncertainty (e.g. confidence intervals)
☐	☒	For null hypothesis testing, the test statistic (e.g. *F*, *t*, *r*) with confidence intervals, effect sizes, degrees of freedom and *P* value noted<br>*Give P values as exact values whenever suitable.*
☒	☐	For Bayesian analysis, information on the choice of priors and Markov chain Monte Carlo settings
☒	☐	For hierarchical and complex designs, identification of the appropriate level for tests and full reporting of outcomes
☒	☐	Estimates of effect sizes (e.g. Cohen's *d*, Pearson's *r*), indicating how they were calculated

Our web collection on statistics for biologists contains articles on many of the points above.

Software and code

Policy information about availability of computer code

Data collection	Nikon Elements Br software (version 4.30.01)<br>Sciex Analyst Chromatography Software (version 1.7.1)<br>Agilent ChemStation Software (B.04.01 LC 1200)<br>DeNovix® DS-11 Spectrophotometer software (version v 4.1.9)<br>Zeiss ZEN software (version 1.0, 2011)
Data analysis	GraphPad prism (version 9)<br>Microsoft Excel (2021, version 16.57)<br>ImageJ 1.53k, (Java 1.8.0_172 [64-bit])<br>ImageJ/Fiji (version 2.1.0/1.53f)<br>BioRender.com (2022)

For manuscripts utilizing custom algorithms or software that are central to the research but not yet described in published literature, software must be made available to editors and reviewers. We strongly encourage code deposition in a community repository (e.g. GitHub). See the Nature Portfolio guidelines for submitting code & software for further information.

Data

Policy information about availability of data

All manuscripts must include a data availability statement. This statement should provide the following information, where applicable:

- Accession codes, unique identifiers, or web links for publicly available datasets
- A description of any restrictions on data availability
- For clinical datasets or third party data, please ensure that the statement adheres to our policy

The authors declare that all data supporting the findings of this study are available within the paper and its supplementary information files. Source data are provided with this paper.

Field-specific reporting

Please select the one below that is the best fit for your research. If you are not sure, read the appropriate sections before making your selection.

- [x] Life sciences
- [ ] Behavioural & social sciences
- [ ] Ecological, evolutionary & environmental sciences

For a reference copy of the document with all sections, see nature.com/documents/nr-reporting-summary-flat.pdf

Life sciences study design

All studies must disclose on these points even when the disclosure is negative.

Sample size	No statistical methods were used to predetermine sample size. For in vitro experiments (in vitro release, SEM analysis), n=3 implants were analyzed per time point, n=4 BALB/c mice were used for PK analysis per formulation. For efficacy studies, sample size was n=6 per group. We performed sample size calculations with our experimental data and determined that 6 mice per group was sufficient to detect significant differences in CFU with 0.99 power (alpha =0.05)
Data exclusions	No data were excluded from the analysis
Replication	All in vitro experiments were done at least in triplicates. When possible, at least 4 mice were used for PK analysis. The PK of the optimized formulation was repeated in 3 independent experiments (Figures 3b,c,e). Efficacy of the formulation was assess in 2 different experiments, pre-exposure prophylaxis (Figure 6a-c) and post-exposure treatment (Figure 6 d-h). All attempts at replication were successful.
Randomization	PK and efficacy studies: animals were assign to the experimental groups by simple randomization. In vitro analysis of solidified implants (in vitro release rates, SEM analysis): For each experiment, implants were created in vitro by injection to PBS. All created implants from each formulation were analyzed.
Blinding	Investigators were not blinded during in vitro release studies, all created implants from each formulation were analyzed. Investigators scanning micrographs of implants using SEM were blinded to the experimental group allocations. Investigators were not blinded for group allocation for formulation administration to animals due to color differences of the drug formulation and placebo. Investigators collecting samples for PK analysis were blinded to experimental group allocation or type of treatment. Investigators analyzing drug content (LC/MS) were blinded to the experimental group allocations. Investigators analyzing data were not blinded for the group allocation.<br>In the prevention efficacy study, investigators involved in Mtb exposures, sample collections and sample analyses (CFU assay) were blinded to the experimental group allocations. Investigators analyzing lung sections by IHC in efficacy studies were not blinded to group allocation due to apparent differences in tissue pathology between treated and untreated groups without IHC staining (Figure 1f), however, images were obtained and evaluated from all mice in all treatment and placebo groups.

Reporting for specific materials, systems and methods

We require information from authors about some types of materials, experimental systems and methods used in many studies. Here, indicate whether each material, system or method listed is relevant to your study. If you are not sure if a list item applies to your research, read the appropriate section before selecting a response.

Materials & experimental systems

n/a	Involved in the study	
x		Antibodies
x		Eukaryotic cell lines
x		Palaeontology and archaeology
	x	Animals and other organisms
x		Human research participants
x		Clinical data
x		Dual use research of concern

Methods

n/a	Involved in the study	
x		ChIP-seq
x		Flow cytometry
x		MRI-based neuroimaging

Animals and other organisms

Policy information about studies involving animals; ARRIVE guidelines recommended for reporting animal research

Laboratory animals	Female 10-15 week old BALB/cJ (The Jackson Laboratory, Bar Harbor, ME) mice were used. MIce were housed in SPF animal facility at 20-23 C, 30-70% humidity and 12h dark/light cycle.
Wild animals	The study did not involve wild animals.
Field-collected samples	The study did not involve samples collected from the field.
Ethics oversight	Animal studies were carried out according to protocols approved by the Institutional Use and Care Committee at the University of North Carolina-Chapel Hill and in adherence to the National Institutes of Health Guide for the Care and Use of Laboratory Animals.

Note that full information on the approval of the study protocol must also be provided in the manuscript.
